# Rigidity and Flexibility in Rotaxanes and Their Relatives; On Being Stubborn and Easy-Going

**DOI:** 10.3389/fchem.2022.856173

**Published:** 2022-04-07

**Authors:** Rachel E. Fadler, Amar H. Flood

**Affiliations:** Department of Chemistry, Indiana University, Bloomington, IN, United States

**Keywords:** conformations, flexible, host-guest chemistry, macrocycle, polyrotaxane, pseudorotaxane, rigid, rotaxane

## Abstract

Rotaxanes are an emerging class of molecules composed of two building blocks: macrocycles and threads. Rotaxanes, and their pseudorotaxane and polyrotaxane relatives, serve as prototypes for molecular-level switches and machines and as components in materials like elastic polymers and 3D printing inks. The rigidity and flexibility of these molecules is a characteristic feature of their design. However, the mechanical properties of the assembled rotaxane and its components are rarely examined directly, and the translation of these properties from molecules to bulk materials is understudied. In this *Review*, we consider the mechanical properties of rotaxanes by making use of concepts borrowed from physical organic chemistry. Rigid molecules have fewer accessible conformations with higher energy barriers while flexible molecules have more accessible conformations and lower energy barriers. The macrocycles and threads become rigidified when threaded together as rotaxanes in which the formation of intermolecular interactions and increased steric contacts collectively reduce the conformational space and raise barriers. Conversely, rotational and translational isomerism in rotaxanes adds novel modes of flexibility. We find that rigidification in rotaxanes is almost universal, but novel degrees of flexibility can be introduced. Both have roles to play in the function of rotaxanes.

## Graphical Abstract

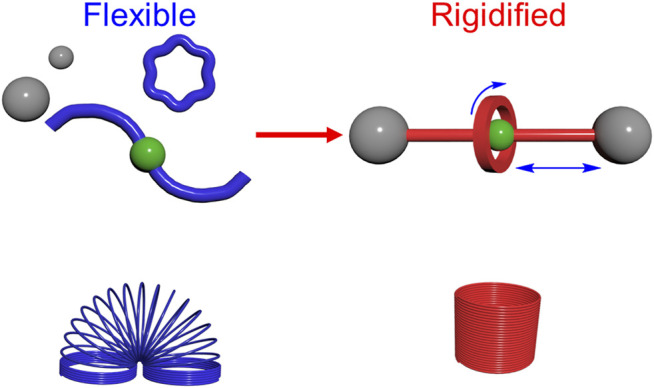

## Introduction

**FIGURE 1:**
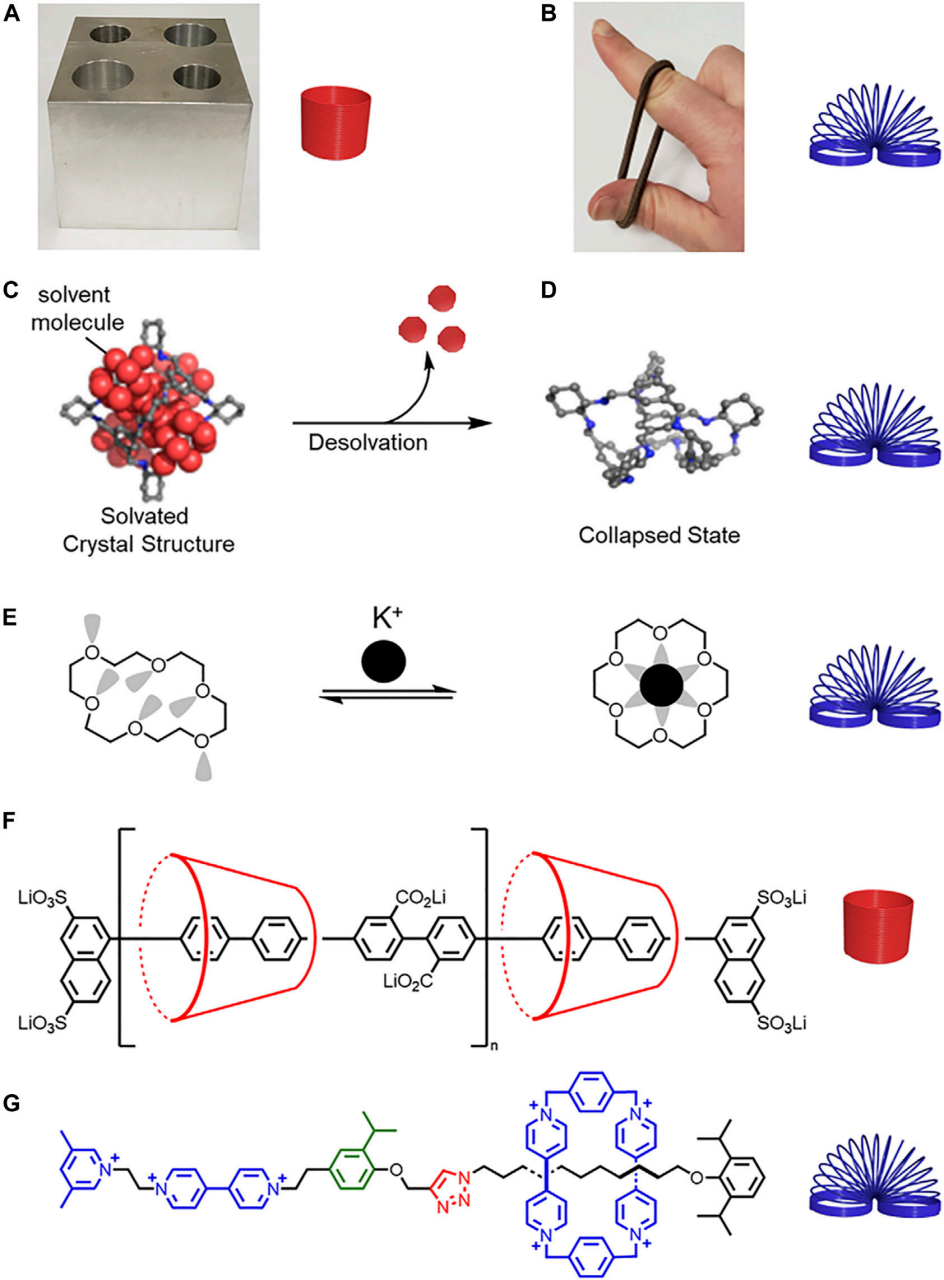
**(A)** Hard metal block and rigid spring. **(B)** Soft rubber band and flexible spring. **(C)** Schematic representation of a solvated molecular cage **(D)** collapsing upon desolvation. Adapted with permission from Ref. (Liu et al., 2014). Copyright 2014 American Chemical Society. **(E)** Structure of a collapsed crown ether that changes shape and rigidifies upon potassium complexation. **(F)** A more rigid polyrotaxane composed of cyclodextrin and a conjugated thread, and **(G)** a less rigid rotaxane composed of cyclobis (paraquat-*p*-phenylene) (CBPQT^4+^) and a thread composed of flexible alkyl chains and rigid aryl building blocks.

Rigidity and flexibility are properties of matter. On the macroscale, these mechanical characteristics are routinely examined using, e.g., stress-strain curves, and they can be quantified by their Young’s modulus, e.g., 10^9^–10^12^ Pa for hard and rigid materials (Figure 1A) and 10^4^–10^9^ Pa for soft and flexible ones (Figure 1B). (Rus and Tolley, 2015) Chemists borrowed many of these concepts to describe similar, albeit difficult-to-measure (Bowser et al., 2021; Zeng et al., 2021), phenomena at the molecular level. Despite this analogy, mechanical properties play out differently on the nanometer-scale of molecules. Starting with macroscale objects, whether soft or hard, they are typically invariant in size or shape until acted upon by some force. For molecules, rigidity and flexibility typically arise from the relative ease of internal motions that allow them to exchange between conformations. These movements are subject to constant random Brownian motion such that the relative populations of different conformations and their rates of interconversion can be quite varied. This perspective informs the examples laid out in this *Review*.

### Rigidity and Flexibility in Macrocycles, Threads, and Assemblies: The Twilight Zone

Looking beyond the molecule, we see rigidity expressed with host-guest complexes in the concepts of preorganization (Doxsee et al., 1987), shape-persistence (Zhang et al., 1994), and collapsibility (Jelfs et al., 2011). Preorganization describes the benefits of affinity and selectivity for guest binding when the receptor has a favorable conformation prior to complexation (Cram, 1986). Preorganization is often associated with the macrocyclic effect. Therein, macrocycle cavities are more preorganized than their acyclic oligomer analogues to produce greater affinities and selectivities (Cram et al., 1981). Preorganized receptors are often described as rigid (Dobscha et al., 2016). Shape-persistence is a newer description that has been ascribed to macrocycles with low conformational freedom (Zhang et al., 1994), and more broadly, with receptors that have minimal shape changes when subjected to an external stimulus such as guest binding (Dobscha et al., 2016; Liu et al., 2016). Conversely, flexibility is associated with large conformational changes and often facilitates collapsibility (Figures 1C,D). (Liu et al., 2014) In the context of macrocycles, collapsibility is commonly associated with flexible receptors where the preferred conformation of the unbound macrocycle collapses into the cavity to some extent. [18]Crown-6 is a classic example where electronic repulsions between the lone pairs on the oxygens and attractive CH•••O interactions from one side of the macrocycle to the other cause the structure to collapse upon itself (Figure 1E). (Steed and Atwood, 2009) More recently, collapsibility has been considered for porous cages and is associated with the removal of solvent that results in a conformational change to a non-porous structure (Holst et al., 2010).

Recent studies on porous cages (Mitra et al., 2013) and macrocycles (Liu et al., 2016) blur the distinctions between rigidity and flexibility in the context of collapsibility and shape-persistence. Cooper and Jelfs examined the collapsibility (Liu et al., 2014) of large porous organic cages (Hasell and Cooper, 2016; Bennett et al., 2020) using computational studies. Jelfs concludes that “rigidity and flexibility are not absolutes; whilst some flexibility and motion of a component is inevitable, excessive flexibility in the linkers is linked to lower product yields (Zhang and Mastalerz, 2014) and increases the likelihood that the end assembly will not be shape persistent” (Santolini et al., 2017) and will collapse to form non-porous structures. For macrocycles, Flood, Singharoy, and Raghavachari found that shape-persistent macrocycles do not necessarily require a small number of accessible conformations, rather that all of the accessible conformations must have similar shapes (Liu et al., 2016). Shape-similar conformers were observed with shape-persistent macrocycles, including Flood’s triazolophane (Ramabhadran et al., 2014) and cyanostar macrocycles (Liu et al., 2016). As a result, we can expect rigidity and flexibility for rotaxanes to lie on a similar spectrum.

**FIGURE 2:**
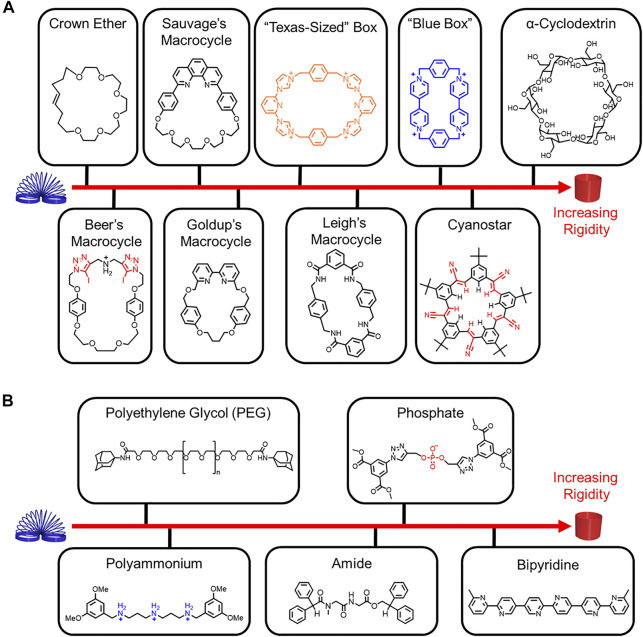
Relative rigidity of representative **(A)** macrocycles and **(B)** threads.

Rigidity is associated with fewer thermally accessible conformations and co-conformations as well as higher energy barriers, while flexibility is associated with greater conformational flexibility and lower energy barriers (*vide infra*). Conformational freedom can be used as a framework to understand rigidity and flexibility in rotaxane architectures. Despite our best intensions, conformational freedom is not often calculated, and thus assessments of rigidity and flexibility are often made in the comparison of two molecules. Rigidity and flexibility can also be determined based on the composition of the macrocycles, the threads, and any changes that occur upon threading. For example, a polyrotaxane composed of cyclodextrin and a conjugated thread (Michels et al., 2003) (Figure 1F) is more rigid than a molecular pump where the thread contains rigid and flexible functional groups and the cyclobis(paraquat-*p*-phenylene) (CBPQT^4+^) macrocycle (Cheng et al., 2015) (Figure 1G). To give a feel for the relative rigidity and flexibilty of rotaxane building blocks in this *Review*, we ranked a collection of macrocycles (Figure 2A) and threads (Figure 2B), and we use our understanding of their rigidity to help define the conformational space of the resulting rotaxanes.

### Conformational Space of Rigid and Flexible Rotaxanes

**FIGURE 3:**
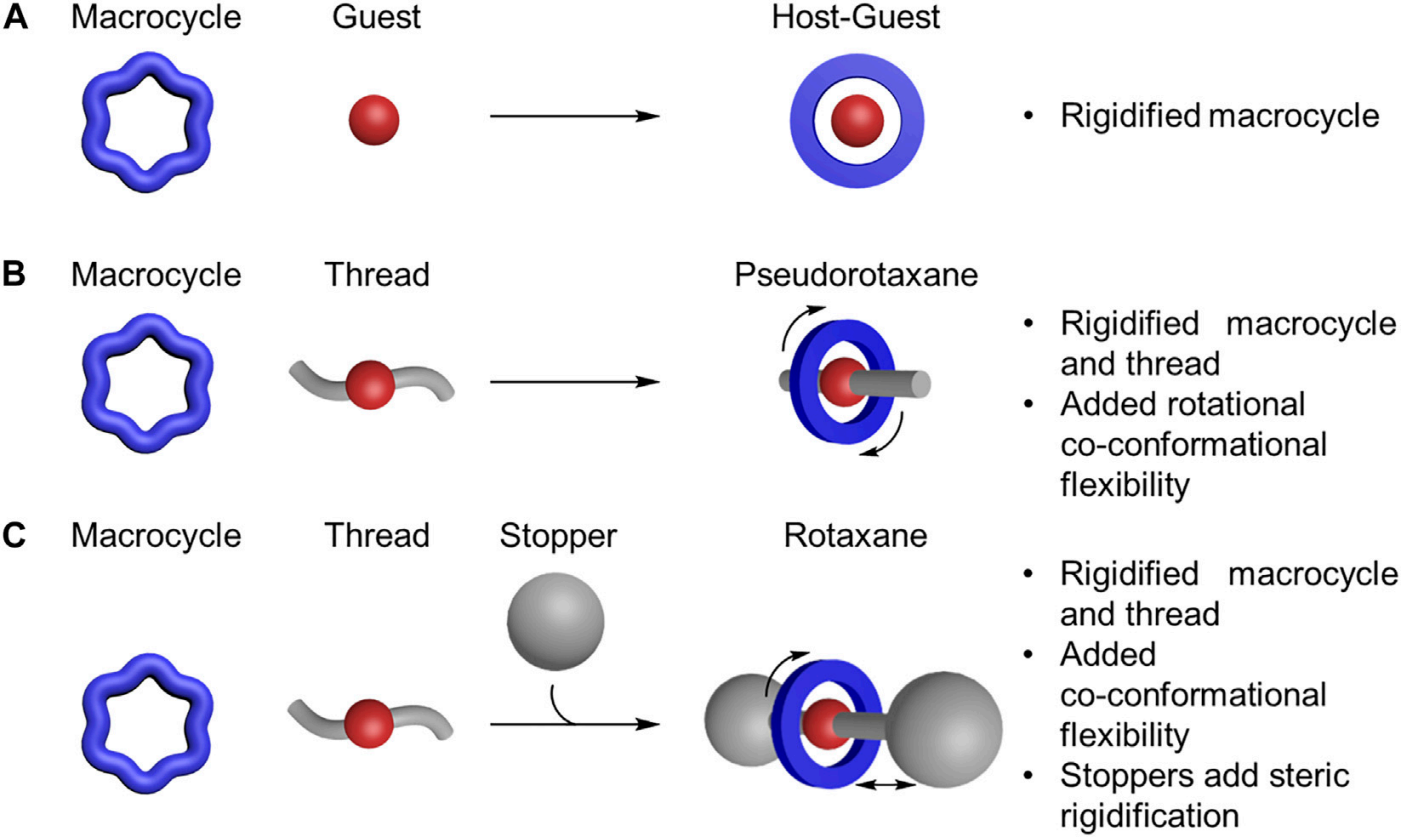
Formation of **(A)** host-guest complex, **(B)** pseudorotaxane, and **(C)** rotaxane from macrocycles, guests, threads, and stoppers.

The rigidity and flexibility of host-guest complexes (macrocycle and guest, Figure 3A), pseudorotaxanes (macrocycle and thread, Figure 3B) and rotaxanes (macrocycles on a stoppered thread, Figure 3C) are more than the sum of their parts. Beyond their intrinsic conformational properties, the rigidity of macrocycles and threads is usually enhanced upon threading on account of both components adopting preferred geometries that stabilize one set of conformations over another. Conversely, flexibility is also introduced in the form of rotational, translational, and other forms of co-conformational isomers (Sevick and Williams, 2014). Co-conformational isomers refer to the two or more preferred locations of a macrocycle along or around a thread (Neal and Goldup, 2014). These co-conformations are regulated by interactions between the macrocycles and threads in the interlocked architecture. For example, steric interactions between a stopper and the macrocycle can limit translational co-conformations (Figure 3C), (Qiao et al., 2016) further rigidifying the architecture. For all these reasons, a fundamental understanding of the rigidity and flexibility in the individual components and of the resulting rotaxane is essential for describing their mechanical properties.

**FIGURE 4:**
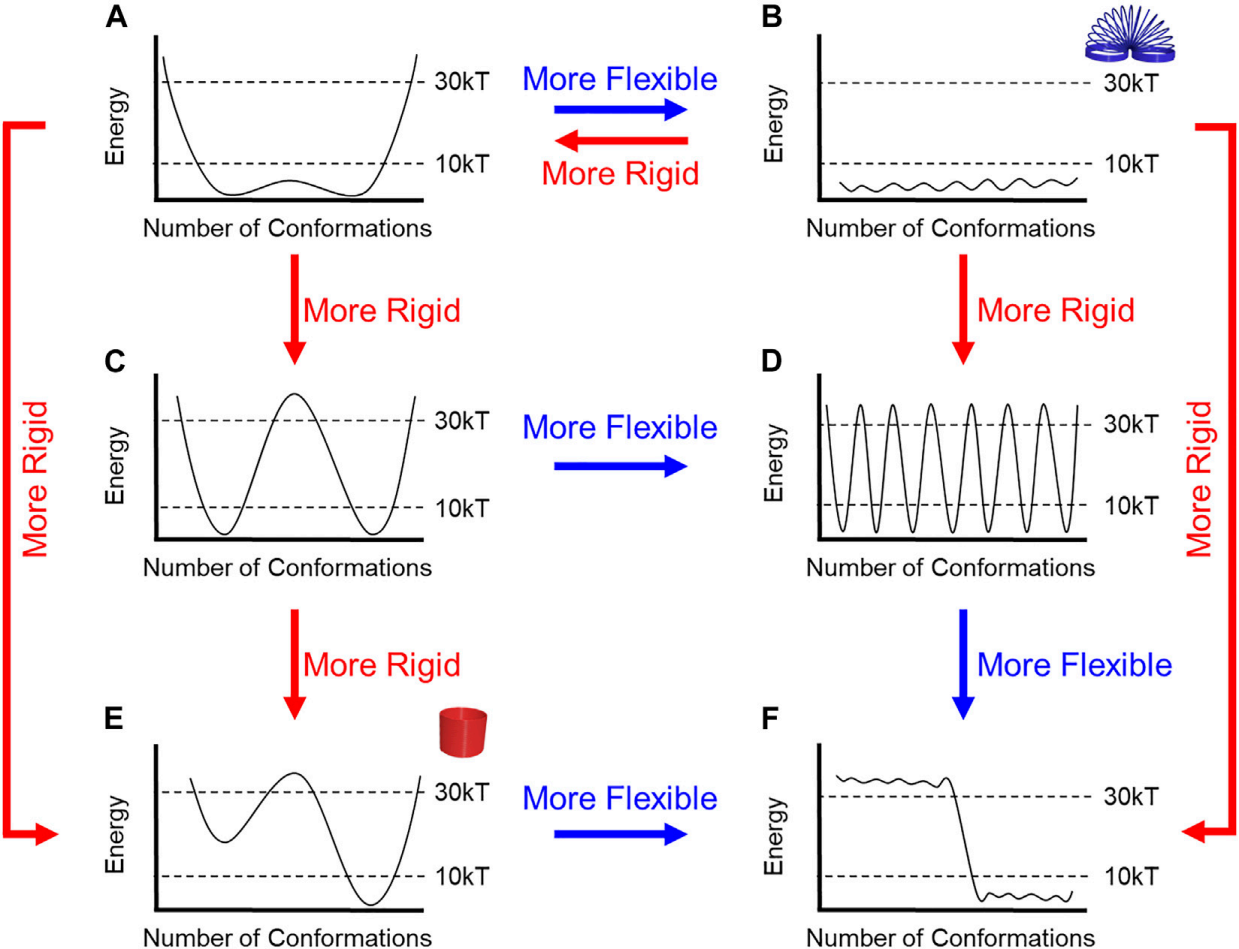
**(A–F)** Flow chart to describe relative rigidity and flexibility for energy diagrams based upon barrier heights and the number of energetically accessible conformations.

The mechanical properties defined by the number of conformations and the height of the barriers (Figure 4) can differ dramatically from the most rigid (Figure 4E) to the most flexible (Figure 4B). Some rotaxanes will be more or less flexible than others. E.g., a rigid molecule might have (Figure 4C) two accessible conformations with high energy barriers (>30 kT) while a molecule with eight conformations (Figure 4B) and low barriers (< 10 kT) will be more flexible.

Herein, our selections of kT are defined relative to the observations that can be made using NMR spectroscopy, which is typically used to characterize the structures of interlocked molecules. We selected 30 kT as a convenient cutoff for a barrier that will produce a rotaxane with dynamics occurring slower than the NMR timescale. Nevertheless, it remains an accessible barrier that can be overcome at room temperature. Here, 30 kT corresponds to 20 kcal/mol (84 kJ/mol) with a half-life of approximately 1 minute based on the Eyring equation. We selected 10 kT (6 kcal/mol, 25 kJ/mol) as a barrier that is substantially lower than the NMR timescale. The 10 kT is also a thermodynamic cutoff such that any conformations sitting above this level will not be populated at room temperature based on Boltzmann population statistics.

Evaluating the energetic accessibility of the conformations (and co-conformations) is crucial to defining rigidity. Different conformations may be present, but if their energies are too high (>10 kT), then they do not contribute to the Boltzmann populations and should not be considered when evaluating a molecule’s relative rigidity. For instance, if half of the molecule’s possible conformations are not thermally accessible, then the molecule will be more rigid, e.g., compare Figures 4A,E and Figures 4B,F.

The ability to overcome energy barriers is also critical to evaluating rigidity, especially when barrier heights suggest rigidity and the number of conformations suggest flexibility, and vice versa. When a molecule has multiple low-energy conformations (Figure 4D) but with high barriers between them, the molecule would be considered more rigid than a different molecule with a similar number of low-energy conformers and low energy barriers (Figure 4B). In the case (not shown) when each conformation is separated by large barriers (>>30 kT), they cannot rapidly interconvert at room temperature, and these conformations are closer to configurational isomers (Anslyn and Dougherty, 2006) or atropisomers (Cheng et al., 2021). Alternatively, a molecule with just a few conformations (Figure 4C), which would lend it rigidity, can be considered more flexible when the barriers between conformations are smaller (Figure 4A).

### Overview

We will outline the rigidity and flexibility of a rotaxane’s building blocks and consider how they change when forming threaded architectures. We will discuss how intermolecular and steric interactions regulate the conformational freedom of macrocycles and threads to form rigid or flexible rotaxane architectures (Figure 4). Specifically, we will show that intermolecular interactions mediating host-guest recognition at the primary binding site (Li et al., 2018) can rigidify the components and thus the overall assembly. In addition, secondary macrocycle-thread (Clegg et al., 1999) and macrocycle-macrocycle (Belowich et al., 2012) interactions have an impact. These can originate from noncovalent contacts (stabilizing) or from sterics (destabilizing). In addition, we will show how the macrocycle can access additional translational and rotational co-conformations along the thread thereby introducing novel forms of flexibility (Sevick and Williams, 2014). Other threaded and interlocked structures, such as catenanes, will also display many of these concepts, but we have focused on linear structures in the interest of brevity. Similarly, illustrative examples have been selected rather than providing exhaustive coverage of the literature. Strategies to rigidify individual macrocycles and threads based on covalent bonding have already been extensively described by others (Greenaway et al., 2019; Turcani et al., 2019; Bennett et al., 2021) and is outside the scope of this *Review*. We will end by providing examples of how rotaxane rigidity and flexibility can play a role in molecular technologies including molecular switches, molecular machines, and bulk materials at the macroscopic scale.

## Rigidity and Flexibility in Rotaxanes With Rigid Ligands for Complementary Metals

We start by considering the use of rigid components that have relatively well-defined structures (Zhao et al., 2009; Ueda et al., 2017). By combining conformationally rigid ligands together with complementary metals that display distinct binding geometries (Fujita et al., 1990; Lavendomme et al., 2019), rigidity can be increased in higher-order assemblies, i.e., cages (Dalton et al., 2015; Carpenter et al., 2019) and polyhedral (Fujita et al., 2016), as well as in rotaxanes. The conformational space needs to be considered collectively and individually to evaluate rotaxane rigidity and flexibility.

**FIGURE 5:**
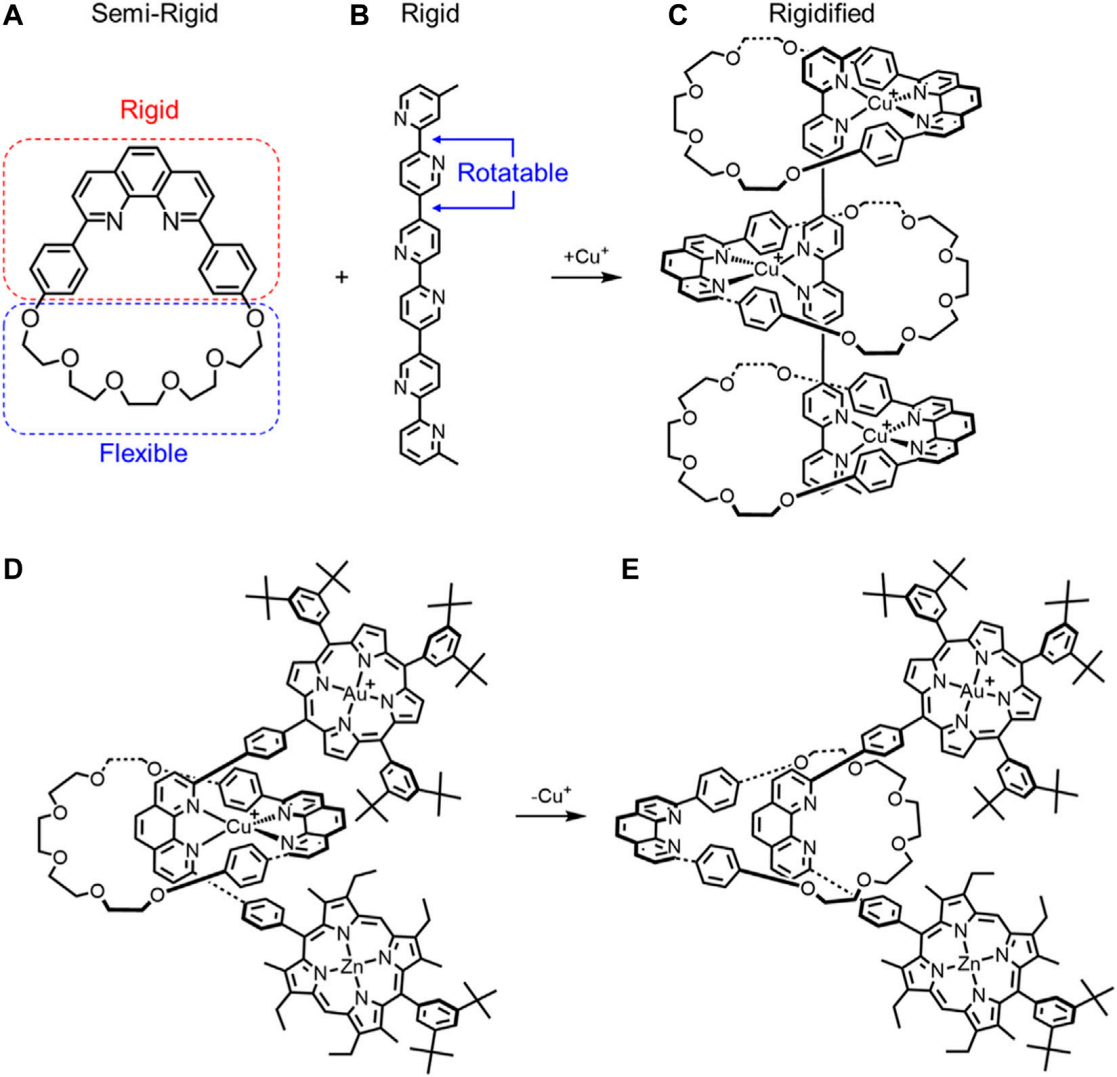
**(A)** Semi-rigid macrocycle and **(B)** rigid thread combine to make a **(C)** rigidified pseudorotaxane upon Cu^+^ complexation. A rotaxane composed of a semi rigid macrocycle and rigid thread have different macrocycle rotational conformations upon **(D)** metal complexation and **(E)** decomplexation.

In 1995, Lehn combined three macrocycles (Figure 5A) with one thread (Figure 5B) to create a metal-templated rigid-rack pseudorotaxane architecture (Figure 5C). (Sleiman et al., 1995) The macrocycle used is semi-rigid (Figure 5A) and is composed of two halves, a rigid diphenyl-phenanthroline core (red, Figure 5A) and a flexible glycol chain (blue, Figure 5B). Upon formation of the pseudorotaxane by threading the ligand inside the macrocycles, the conformational freedom of the glycol chains decreases, and they become rigidified. In this case, the number of thermally accessible conformations for the glycol chain will likely decrease (e.g., Figure 4B →Figure 4F). Furthermore, the two phenyl rings on the phenanthroline portion will have two degenerate conformations. While these conformations are expected to remain equi-energetic, the barrier between them is expected to increase relative to the unbound state (e.g., Figure 4A →Figure 4C). For the uncomplexed thread, the bipyridine subunits are also relatively rigid, but rotations are still possible about the five single bonds (Figure 4A). This rotation is critical for the thread to be able to adopt a geometry suitable for metal-directed threading of the macrocycle. In the pseudorotaxane, three of the five rotatable bonds become fixed, thereby reducing the number of accessible conformations to further increase the rigidity of the threaded architecture (e.g., Figure 4A→Figure 4E). In sum, both components are rigidified upon formation of the threaded architecture.

In 1993, Sauvage created a porphyrin-stoppered [2]rotaxane (Figure 5D). (Chambron et al., 1993) In addition to the semi-rigid macrocycle, the thread is highly rigid with only four rotatable bonds along its length. The rotational freedom of the phenyl rings encased by the rotatable bonds is expected to be lowered in the rotaxane architecture further rigidifying the rotaxane, e.g., Figure 4A→Figure 4C. Copper complexation also fixes the relative location of the macrocycle into one preferred rotational co-conformation and one preferred translational co-conformation. Upon demetallation, the primary interaction at the binding site is removed leading to an increase in overall flexibility (Figure 5E). The preferred rotational co-conformation of the macrocycle changes such that the phenanthroline binding site points away from the porphyrin stoppers. In the absence of copper complexation fixing the macrocycle in one location, we also expect increasing rotational freedom in the single bonds and for greater co-conformational freedom to enhance flexibility (Figure 4E→Figure 4F).

**FIGURE 6:**
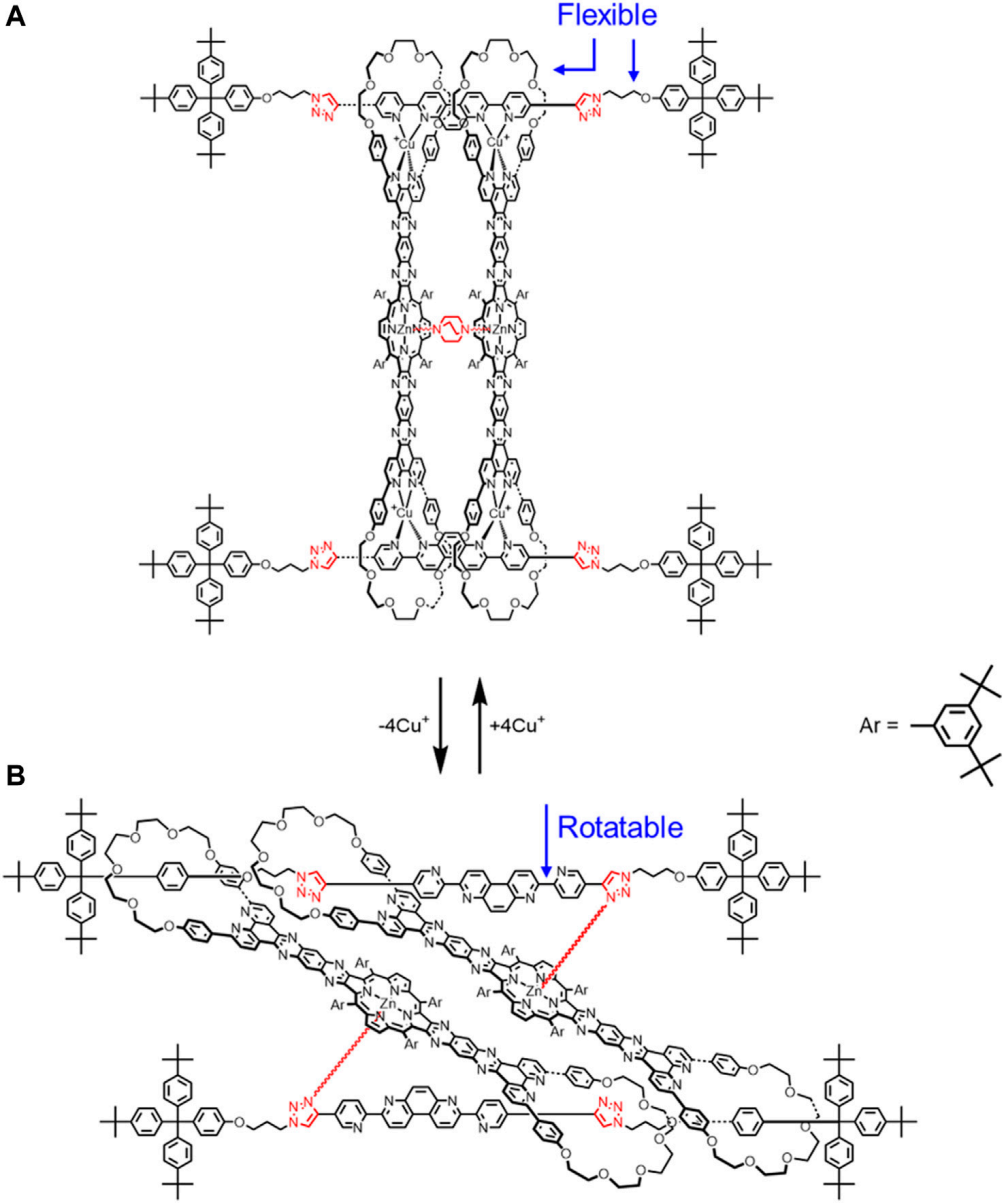
Cyclic [4]rotaxane switch with two translational states upon **(A)** Cu^+^ complexation and **(B)** decomplexation.

Sauvage also created a new class of cyclic [4]rotaxane that is composed of two highly rigid rods threaded through two semi-rigid bis-macrocycles (Figure 6A). (Collin et al., 2010) There are only a few flexible parts. These include alkyl linkers on the thread, the glycol chains on the macrocycles, and the rotatable bonds on the thread. The guest molecule DABCO (1,4-diaxabicyclo[2.2.2] octane) can be accommodated between the two metal centers on the porphyrin plates (Figure 6A). Overall, guest recognition in combination with copper coordination results in a rigid rotaxane conformation. Upon Cu^I^ demetallation of the macrocycles, this conformation is no longer energetically favored, and the rotaxane collapses. New intramolecular interactions form between the triazoles and Zn^II^ ions that help to stabilize a different two-fold degenerate co-conformation where guest recognition is not possible (Figure 6B). In the demetallated rotaxane, interactions between the triazole and the zinc will introduce rigidity, but the pyridines in the thread can more freely rotate than the metalated rotaxane, which will allow more rotational co-conformations to be accessed. As a result, we can say that the demetallated rotaxane will be *more* flexible than the metalated one (Figure 4E → Figure 4F). The term *more* is especially critical here, as the rotaxane is still quite rigid, even with this added degree of translational flexibility.

## Rotaxane and Pseudorotaxane Rigidification Through Intermolecular Interactions at a Primary Binding Site

In this section, we focus on the combination of flexible components, and we examine the use of intermolecular contacts to rigidify the rotaxanes. We will also examine designs that stabilize rotational co-conformations using guest recognition to enhance rigidity.

**FIGURE 7:**
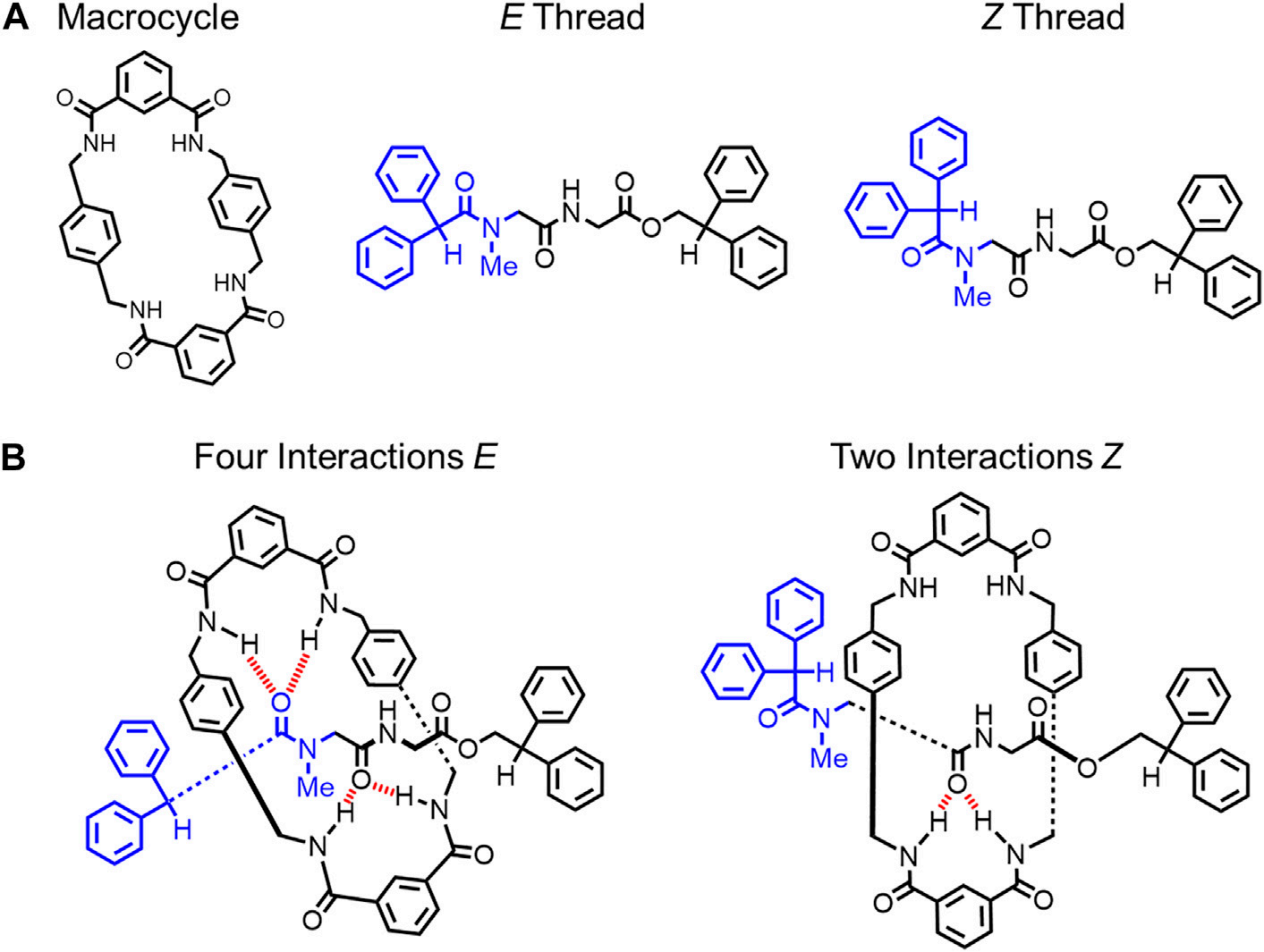
**(A)** Macrocycle structure, thread conformations and **(B)** hydrogen-bond interactions in the two rotaxane architectures.

In 1999, Leigh reported an amide-based rotaxane where rigidification is controlled by changing the hydrogen-bonding network at the primary binding site by using solvent (Figure 7). (Clegg et al., 1999) The rotaxane is composed of a single thread that can access *E* and *Z* rotamers of the amide (blue, Figure 7A). (Clegg et al., 1999) In this [2]rotaxane, a hydrogen-bonding network forms between complementary amide groups on the macrocycle and the thread (Figure 7B). (Clegg et al., 1999) The thread’s *E* rotamer forms four hydrogen bonds while the *Z* rotamer can only form two. When dimethylsulfoxide (DMSO) is used as the solvent, the hydrogen bonding between the thread and the macrocycle is broken. As a result, there is no preference for the *E* or the *Z* rotamer, and both rotamers are observed by ^1^H NMR spectroscopy in solution. This data indicates that the rotaxane has two accessible conformations. However, by using tetrachloroethane as a non-polar solvent, the hydrogen bond network is recovered, and the *E* rotamer with four hydrogen bonds was favored by 4–6 kcal mol^−1^ (Figure 7B). This *E* thread conformation was the only one observed in solution. As a result, the hydrogen-bond network stabilized one dominant thread conformation over the other (Figure 4C→Figure 4E) and created a more rigid structure.

**FIGURE 8:**
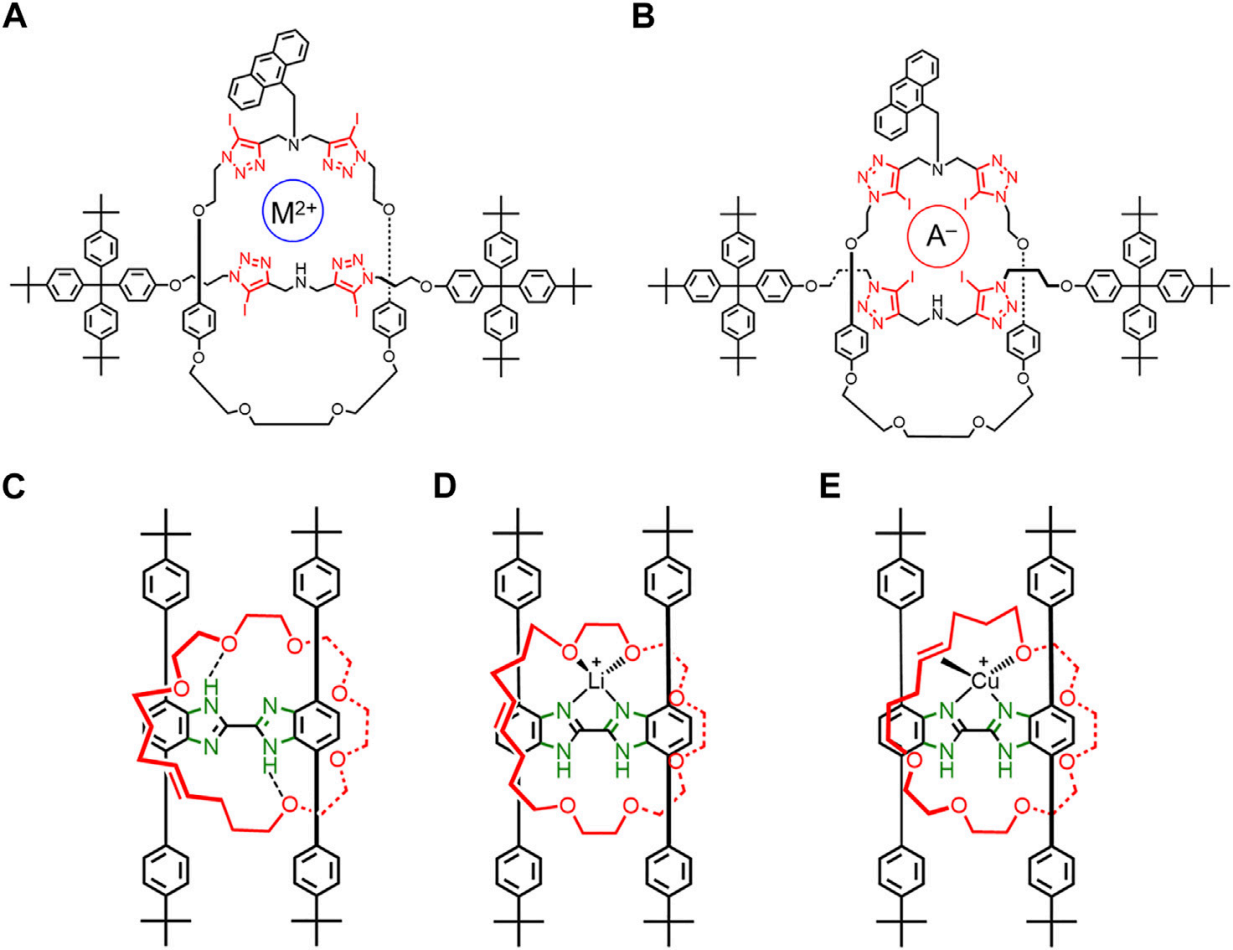
A [2]rotaxane where **(A)** cation and **(B)** anion binding results in different iodotriazole conformations. Rotaxane rotational conformations with H-shaped thread and flexible macrocycle when **(C)** no metal cation is present, **(D)** when Li^+^ is present, and **(E)** when Cu^+^ is present.

In 2018, Beer reported a rotaxane composed of a semi-rigid macrocycle and thread where the conformation of the binding units, in the form of halogen-bonding iodotriazoles, is regulated by the charge of the ionic guest (Figures 8A,B) (Li et al., 2018). Both macrocycle and thread are composed of rigid iodotriazoles and phenyls together with flexible alkoxy linkers. The four iodotriazole rings can undergo endo/exo-dentate flipping, which results in conformations where either the nitrogen lone pairs on the iodotriazoles can bind cations (Figure 8A) or the iodine atoms can bind anions (Figure 8B). The complexation of either a cation or anion selects one geometry over the other. Thus, ion binding results in the rigidification of the rotaxane by fixing the iodotriazole conformations (Figure 4B→Figure 4E). Presumably, ion binding would also restrict the rotational and translation motions of the macrocycle, further rigidifying the assembly. Rotaxane rigidification upon ion binding was examined using an anthracene reporter attached to the macrocycle as a reporter (Li et al., 2018). When anions were bound to the neutral rotaxane, a drastic fluorescence enhancement was observed. This effect was attributed to a decrease in the probability of non-radiative decay, which typically emerges from a more rigid structure.

In 2016, Loeb used a broken symmetry macrocycle to examine rotational co-conformations in rotaxanes. Loeb’s rotaxanes (Figures 8C–E) all have a rigid H-shaped axle and a flexible macrocycle that contain glycol and an alkene functional group that can bind metal cations (Baggi and Loeb, 2016). When no metal cations are present, the macrocycle forms two NH•••O hydrogen bonds between the macrocycle and the thread (Figure 8C). The rotational conformation and dynamics of the macrocycle will likely change upon addition of Li^+^ (Figure 8D, 4B→D) and Cu^+^ (Figure 8E, Figure 4B→Figure 4E). The crystal structures and 2D NMR studies showed that Li^+^ binds two oxygens on the macrocycle (Figure 8D), and Cu^+^ favors binding to one oxygen and the olefin (Figure 8E). An additional Li^+^ conformation was observed in the solid state with three oxygens on the macrocycle bound instead of two. These studies (Figure 8) show that the addition of different guests can result in the rigidification of different rotaxane co-conformations in solution and in the solid state.

**FIGURE 9:**
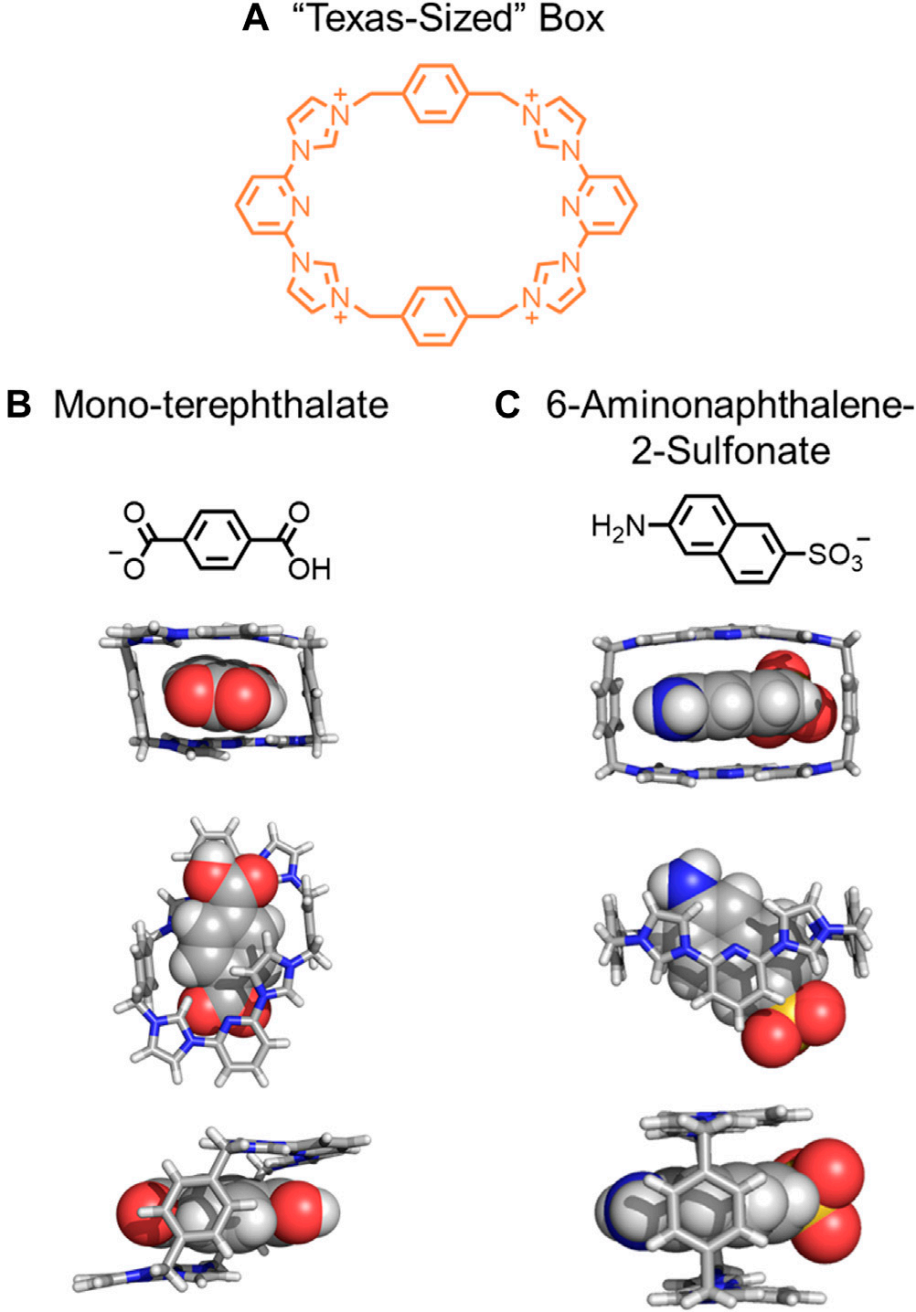
**(A)** The “Texas-sized” box, **(B)** mono-terephthalate guest and resulting chair conformation, and **(C)** 6-aminophthalene-2-sulfonate guest and resulting box-like conformation.

An important concept to expand is that rigidification of macrocycles and threads does not always lead to a single rigid assembly. Rather, a few different rigidified conformations may be observed (Figure 4B→Figure 4D). In 2010, Sessler created a “Texas-sized” molecular box (Figure 9A). (Gong et al., 2010) This macrocycle is more conformationally flexible and less shape-persistent than cyclobis(paraquat-*p*-phenylene) (CBPQT^4+^). The CBPQT^4+^, however, only has a box-like shape, (Odell et al., 1988; Fahrenbach et al., 2012) although it has a few degenerate ring flipping modes (Jang et al., 2005; Fernando et al., 2016; Liu et al., 2018). When the “Texas-sized” box was crystallized with four PF_6_^−^ anions, three different conformations were observed. The flexibility of the “Texas-sized” box enables the macrocycle to adopt different conformations for binding different aromatic threads (Figures 9B,C) to form rigidified pseudorotaxanes (Figure 4B→Figure 4D). (Rambo et al., 2012) Observed conformations include a partial chair (Gong et al., 2011), complete chair (Gong et al., 2011), and two box-like conformations (Gong et al., 2011; Gong et al., 2012). A chair (Figure 9B) and a box-like conformation (Figure 9C) are possible conformations that the “Texas-sized” box has displayed in pseudorotaxanes. Regardless of the observed conformation, complexation helps rigidify the macrocycle and stabilize specific conformations. In these cases, ion-ion, CH‒π and π‒π interactions involving the guest act to rigidify the macrocycle producing rigidified pseudorotaxane architectures. Interestingly, these pseudorotaxanes can also be linked together by hydrogen bonds between carboxylate and carboxylic acid ends of the guests to form supramolecular polymers.

## Intermolecular and Steric Interactions With Two Macrocycles at the Same Primary Binding Site

When multiple macrocycles are present along the thread, inter-macrocycle interactions can emerge to further rigidify the rotaxane assembly. The simplest case involves two macrocycles bound at a single binding site. Examples of this 2:1 binding geometry are relatively rare. One series is the phosphate-templated [3]rotaxanes and [3]pseudorotaxanes composed of two cyanostar macrocycles (Lee et al., 2013). These [3]rotaxanes and [3]pseudorotaxanes contain a balance of stabilizing and destabilizing intermolecular interactions between the cyanostar macrocycles and phosphate threads that regulate rotaxane rigidity and flexibility.

**FIGURE 10:**
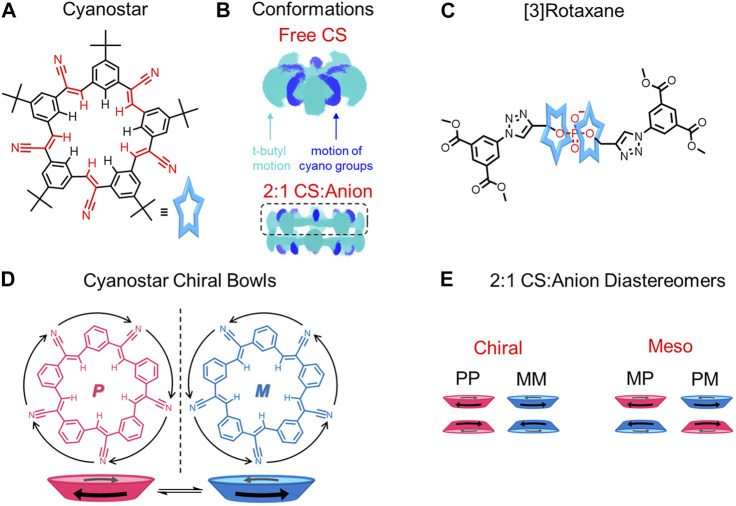
**(A)** Cyanostar macrocycle and **(B)** representations of the molecular dynamics (MD) simulations showing the superposition of conformations of a single (top) and π-stacked dimer of macrocycles (bottom). The top-most macrocycle of the pair (boxed) defines a shallow bowl. The carbon-hydrogen backbone is shown in cyan blue while the cyano nitrogen atoms are colored dark blue. **(C)** [3]rotaxane composed of two cyanostars and an organophosphate dumbbell. **(D)** Top views and cartoon bowl representations of the chiral conformations of cyanostar. **(E)** Chiral and meso combinations of the cyanostar macrocycle dimers. Adapted with permission from (Liu et al., 2016). Copyright 2016 American Chemical Society.

Cyanostar is a shape-persistent anion receptor that, surprisingly, is also flexible (Figure 10A). Experiment-backed computational modeling (Figure 10B) reveals that the cyano-olefins on the macrocycle undergo rocking and rolling motions, which cause the macrocycle to be conformationally flexible (Figure 4B). (Liu et al., 2016) More specifically, 332 conformations are thermally accessible for the free macrocycle (Liu et al., 2016). Nevertheless, all of these conformations have an average 87% shape similarity relative to the most stable conformation such that macrocyclic core of the cyanostar remains largely unchanged (Liu et al., 2016), thus, shape-persistent. The cyanostar was likened to a hummingbird with its rapidly flapping wings corresponding to the rocking cyano-olefins and stationary body to the shape-persistent core. The flexibility of cyanostar introduces the possibility for macrocycle rigidification in threaded architectures (Figure 10A→Figure 10C corresponds to Figure 4B→Figure 4F).

When the cyanostar macrocycle is free in solution, it forms a racemic mixture with the cyano-olefins undergoing rapid conformational exchange (Figure 10B) between *M* and *P* enantiomers (Figure 10D). (Liu et al., 2016) Upon the addition of a disubstituted organophosphate as a thread, two cyanostar macrocycles form a π-stacked dimer around the phosphate binding site and form multiple CH hydrogen bonds (Figures 10B,C). The proximity of the cyanostar macrocycles introduces steric interactions to destabilize those conformations that tilt the cyano-olefins and the *tert*-butyl substituted phenylene rings towards the π-stacked seam (Figure 4B→Figure 4F). In this way, the degeneracy of the bowl-to-bowl interconversion is broken and one of the two macrocycles form either an *M* or *P* enantiomeric bowl (Figure 10D) (Lee et al., 2013; Liu et al., 2016). As a result, dimers form (Figure 10E) as either meso (*MP*) or chiral (*MM*/*PP*) combinations. Overall, both the hydrogen bonding and steric interactions rigidify the cyanostar macrocycles in the threaded architecture.

**FIGURE 11:**
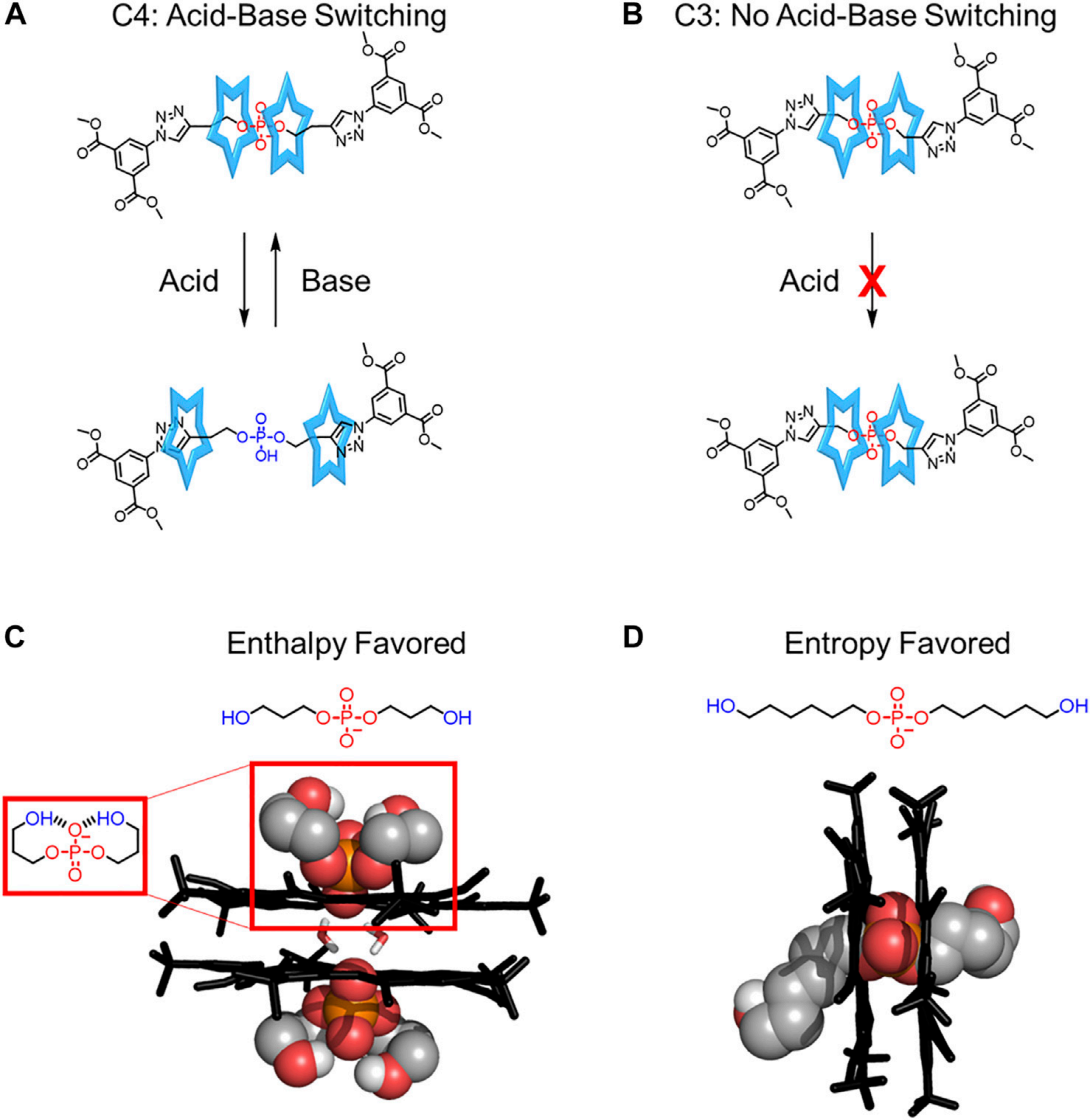
**(A)** Acid-base switching in a more flexible rotaxane and **(B)** the inhibition of switching in a more rigid rotaxane. **(C)** Crystal structures of the cyanostar complexed with dipropanol phosphate in a backfolded conformation and **(D)** dihexanol phosphate in a pseudorotaxane.

Steric interactions between the two cyanostars and the thread also help regulate rotaxane rigidity. In 2016, Flood evaluated the role of steric crowding between the thread’s stopper and two cyanostar macrocycles (Qiao et al., 2016). Two rotaxanes were made that differ in the number of carbons in the linker between the phosphate binding site and the stopper (Figures 11A,B). When the linker is longer, having a four-carbon link, the cyanostars have fewer steric interactions with the stoppers and have reasonable translation freedom (Figure 4A), which enables acid base switching (Figure 11A). When a shorter three-carbon linker is used (Figure 11B), steric interactions between the stopper and the macrocycles prevent cyanostar translation, rigidifying the rotaxane and inhibiting acid-base switching (Figure 4A→Figure 4E for the change in thread from a longer to a shorter linker).

Interactions involving the thread were also shown to regulate the fidelity, and by extension the relative order, of cyanostar-based assemblies (Fadler et al., 2021). Dipropanol phosphate (Figure 11C) is a flexible thread with two terminal hydroxyl groups, ‒OH, that can form intramolecular hydrogen bonds with the phosphate binding site. Crystallography confirmed the formation of a backfolded conformation resulting in a non-covalent ring that binds to the cyanostar in a perched arrangement (Figure 11C) instead of a threaded assembly. To enable pseudorotaxane formation, the length of the alkyl linkers was increased to dihexanol phosphate (Figure 11D) to enhance the number of conformations accessible by the thread. This more flexible thread raised the entropic penalty of competitive ring formation to turn on [3]pseudorotaxane formation (Figure 11D). This study provides a unique example where the flexibility of the thread was increased (Figure 4A→Figure 4B) leading to an increase in the yield of pseudorotaxane formation.

## Rigidifying Oligorotaxanes Through Intermolecular and Steric Interactions

The creation of oligorotaxanes involves multiple macrocycles and multiple binding sites on a thread. Interactions can emerge either between macrocycles or between macrocycles and threads at primary and secondary contact points. However, the introduction of multiple binding sites creates the opportunity for macrocycle-macrocycle interactions to operate across adjacent binding sites.

**FIGURE 12:**
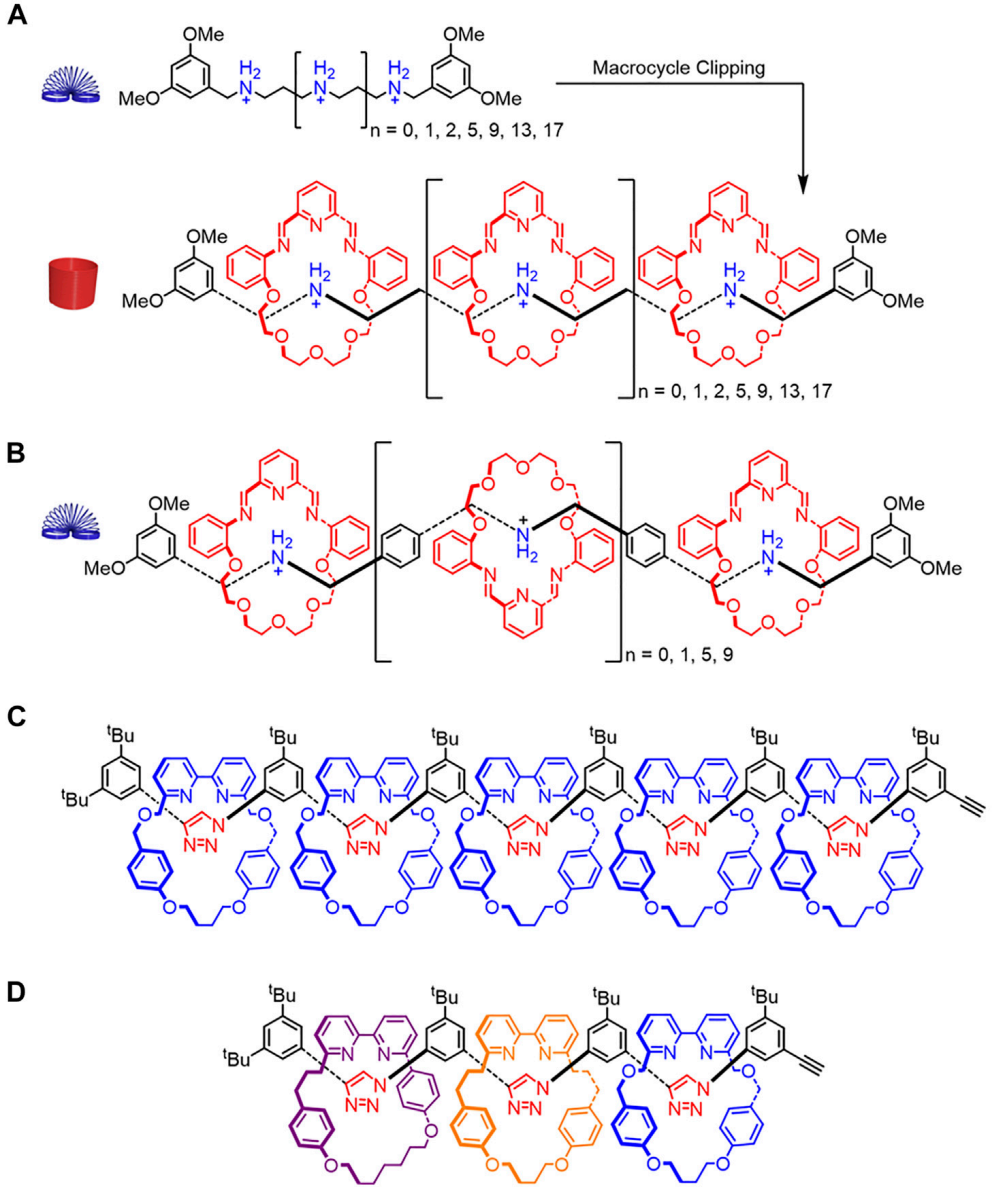
**(A)** Structures of the thread and rigidified rotaxane resulting from π stacking between the semi-rigid macrocycles spaced 3.5 Å apart on a flexible thread. **(B)** A more flexible rotaxane where the distance between binding sites is larger than 3.5 Å and π-π stacking is interrupted. **(C)** Homo[6]rotaxane and **(D)** hetero[4]rotaxane containing rare all-*syn* triazole conformations.

Stoddart showed that π-π interactions between macrocycles at adjacent sites can enhance the rigidity of oligorotaxanes (Belowich et al., 2010; Belowich et al., 2012). Two different rotaxanes composed of the same macrocycle but different threads were created (Figures 12A,B). The macrocycle contains shape-persistent π-surfaces that can form π-π contacts between adjacent macrocycles. Both threads display conformational flexibility, but the distance between the binding sites varies. When the binding sites are 3.5 Å apart (Figure 12A), π-π stacking interactions form between the macrocycles to restrict the conformation of the thread and align the macrocycles defining a rigid, rod-like rotaxane (Figure 4B→Figure 4E). However, when the binding sites are greater than 3.5 Å apart (Figure 12B), no evidence of π-π stacking interactions between adjacent macrocycles is observed. The absence of π-π interactions will produce a rotaxane that is more flexible than the one with π-π stacking (Figure 4B→Figure 4F).

In both cases, rotaxane rigidity was evaluated using a combination of experimental (^1^H NMR, electrospray ionization mass spectrometry, crystal structures) and computational (molecular dynamics) methods. The molecular dynamics simulations were useful in evaluating the structures and resulting rigidity of higher-order oligorotaxanes. Simulations showed that rotational co-conformations produced aligned macrocycles in the more rigid rotaxane architecture (Figure 12A) and disordered ones in the more flexible oligorotaxane architecture (Figure 12B). Furthermore, alignment of the macrocycles caused the rigidified architecture to become curved, which was attributed to electrostatic repulsions between the oxygen lone pairs on adjacent macrocycles (Figure 12A). This bend was not observed when the macrocycles had rotational disorder in the more flexible oligorotaxane (Figure 12B).

Steric interactions between adjacent macrocycles can also restrict the thread’s conformational freedom and lead to more rigidified rotaxanes. In 2016, Goldup reported a rigidified homo [6]rotaxane containing identical macrocycles (Figure 12C) and a sequence-specific hetero[4]rotaxane containing different macrocycles (Figure 12D). (Lewis et al., 2016) The ^1^H–^1^H ROESY, ^1^H NMR spectroscopy, and computational studies suggest that the homo[6]rotaxane displays an all-*syn* triazole conformation (Lewis et al., 2016). This rigidified thread (Figure 4D→Figure 4E) leads to an overall extended rotaxane geometry that has an end-to-end distance of ∼3.8 nm. The ^1^H–^1^H ROESY and ^1^H NMR spectroscopy suggest that the hetero[4]rotaxane adapts an all-*syn* triazole conformation in the thread as well. Overall, Goldup shows how steric interactions between adjacent macrocycles favor one conformation for the thread component to create an extended rotaxane geometry.

**FIGURE 13:**
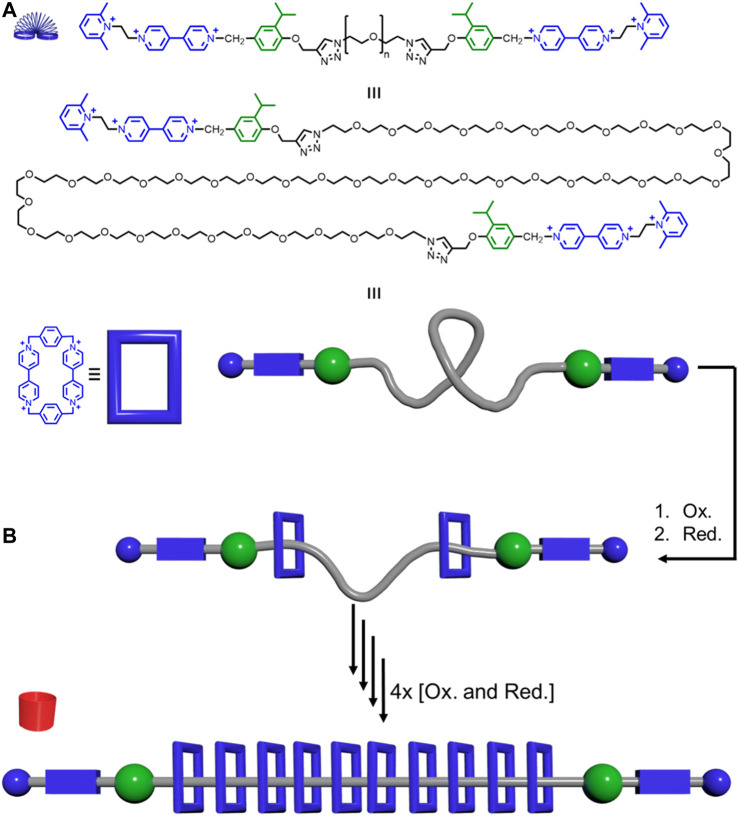
Precise polyrotaxane pump synthesizer: **(A)** Thread structure (top), elongated version (middle), and model together with the structure of the CBPQT^4+^ macrocycle (blue) and its model. **(B)** Pumping of the macrocycles onto the thread generates rotaxanes that get more rigid as more CBPQT^4+^ macrocycles are added.

Electrostatic repulsions between adjacent macrocycles can also help rigidify the overall rotaxane assembly. Stoddart reported a precise polyrotaxane synthesizer where positively charged CBPQT^4+^ macrocycles can be pumped onto the thread (Figure 13A). (Qiu et al., 2020a) This thread has approximately 150 rotatable bonds and is thus highly flexible. Assuming the rotatable bonds have two thermally accessible conformations, this thread will have 2^150^ = 1.4 × 10^45^ conformations. Up to ten positively charged macrocycles can be pumped onto the central polyethylene glycol thread (PEG) portion with a number-average molecular weight (*M*_n_) of 2000 g mol^−1^. These positively charged macrocycles experience electrostatic repulsions. Therefore, increasing the number of macrocycles will elongate and rigidify the PEG backbone (Figure 4B→Figure 4A, Figure 13B). This conformational change in the thread increased the hydrodynamic radius of the rotaxane from 2.7 ± 1.0 to 4.8 ± 1.4 nm. Overall, Stoddart’s system shows how electrostatic repulsions between macrocycles can lead to rigidity in polyrotaxanes.

## Rigidity and Flexibility in Molecular Shuttles and Switches

Diverse components are frequently incorporated into molecular and nanoscale machines (Qiu et al., 2020b; Colasson et al., 2020; Amano et al., 2021), but studies examining the role of rigidity and flexibility in their design (Chen et al., 2021) and operation (Kistemaker et al., 2021) are relatively rare. Exemplary studies involve molecular shuttles (Nygaard et al., 2007; Gholami et al., 2017) and switches (Andersen et al., 2014). Earlier discussions of molecular switches (Figures 5D,E, Figure 6, Figures 11A,B) and machines (Figure 13) placed emphasis on the relative rigidity and flexibility of the various co-conformational states and their thermodynamic preferences. This section will extend the analysis to the roles that barrier heights and kinetics have on rigidity and flexibility.

**FIGURE 14:**
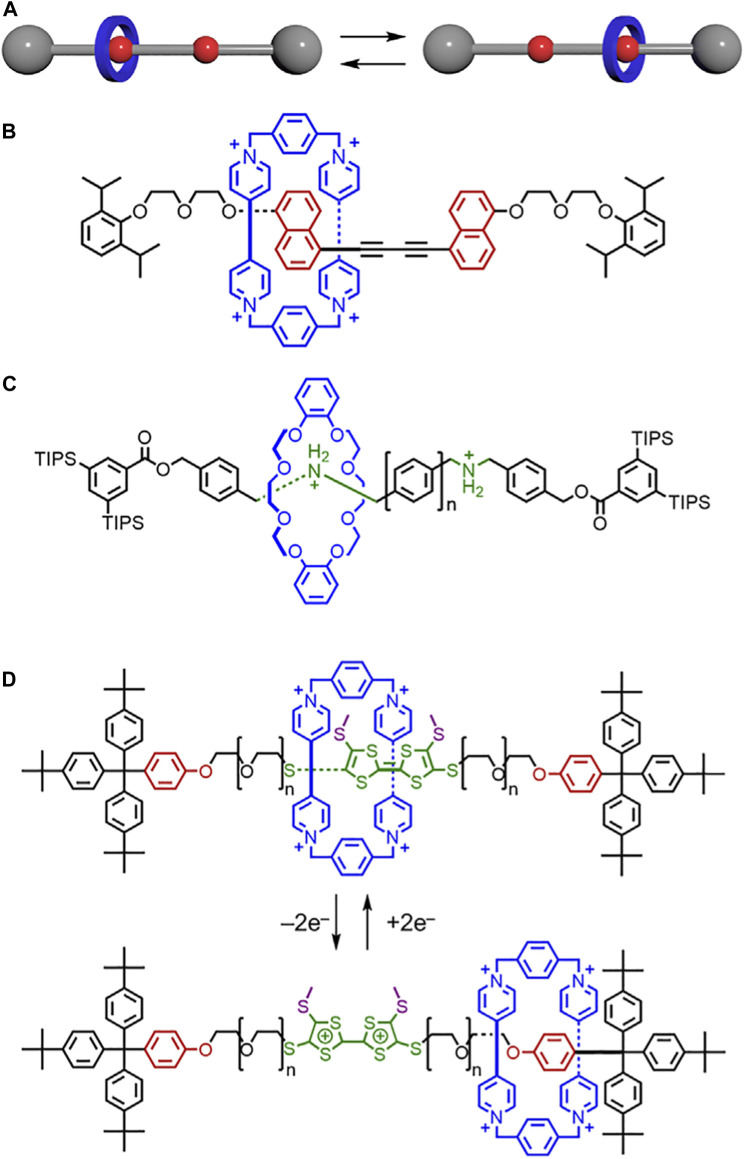
**(A)**Translational motion between isomers in a molecular shuttle. **(B)** Shuttle composed of a CBPQT^4+^ macrocycle and a rigid thread. **(C)** Shuttle composed of a crown ether macrocycle and a thread with rigid phenyl linkers. **(D)** Molecular switch composed of a CBPQT^4+^ macrocycle and a thread with flexible PEG linkers.

The difference between rigid and flexible linkers in molecular shuttles (Figure 14A) was examined by Stoddart in 2007.^63 1^H NMR studies on the rotaxane with degenerate naphthalene sites show dynamic shuttling with a free energy of activation Δ*G*_c_^‡^, of ∼15.0 kcal/mol at ∼240 K when the linkers contain polyethylene glycol (Nygaard et al., 2007; Kang et al., 2004). However, when rigid spacers are used (Figure 14B), the free energy of activation, Δ*G*_c_^‡^, is much lower 9.6 ± 0.1 kcal/mol at 199 K (Nygaard et al., 2007). As a result, the rigid bis-alkyne spacers reduce the energy (Figure 4C→Figure 4A) required to move the macrocycle between binding sites to produce greater translational flexibility of the macrocycle. This difference was attributed to two factors. First, replacing PEG with rigid alkynes decreased the number of secondary CH•••O interactions that can form between the thread and the CBPQT^4+^ macrocycle. This factor reduced the stability of the binding sites. Second, the rigid bis-alkyne linker did not interact with the macrocycle in the same manner as the PEG during movement. This factor reduced the friction. Both effects increase the shuttling rate and extent of translational motion, thus rotaxane flexibility. Rigid building blocks, like alkynes (Michels et al., 2003), are typically associated with higher energy barriers and more rigid architectures (Zhang et al., 1994; Patrick et al., 2022), and this shuttle (Figure 14B) highlights a critical counterexample where lower energy non-covalent interactions lead to lower energy barriers and more translational flexibility.

In 2014, Hirose and Tobe tested the impact of rigidity on the rates of macrocycle shuttling across a series of linker lengths. Rigid linkers were constructed containing different numbers of phenylene spacers (*n* = 1–4, Figure 14C). (Young et al., 2014) Unlike their flexible counterparts (Choi et al., 2006; Deutman et al., 2008; Deutman et al., 2015), the rigid linker lengths do not have a significant impact on shuttling kinetics. However, the barrier to shuttling can be lowered by increasing the polarity of the solvent. These changes were attributed to the solvent’s ability to disrupt hydrogen-bond interactions between the macrocycle and the ammonium binding site. By decreasing the stability of the binding sites, the activation energy to shuttling decreases (Figure 4C→Figure 4A). Thus, the ring’s mobility along the thread increases.

Building on Hirose and Tobe’s work, (Young et al., 2014), Loeb investigated the rates of motion in a [2]rotaxane shuttle with a crown-ether macrocycle and an H-shaped thread in 2017 (Gholami et al., 2017). The H-shaped thread either had one to four phenylene spacers or a napthyl spacer. Unlike Hirose’s and Tobe’s use of a flexible amine binding site, Loeb uses a rigid H-shaped thread. The more rigid thread was anticipated to prevent formation of thread-based conformations that could shorten the length of the track and have unpredictable consequences on shuttling. Loeb’s findings were mostly consistent with Hirose and Tobe such that the length of the rigid linkers did not usually effect shuttling kinetics. However, when linker lengths are short (phenylene *n* = 1), the macrocycle can simultaneously occupy both binding sites, which creates a “short-cut” that lowers the energy barrier to shuttling (Figure 4C→Figure 4A).

Loeb also studied ring-through-ring shuttling with a rigid H-shaped axle bearing two binding sites and two crown ether macrocycles of different sizes (Zhu et al., 2018). When the two macrocycles are too similar in size, each is localized at a single binding site and translational motion was limited, thus creating a more rigid rotaxane. However, when one macrocycle is significantly smaller than the other, ring-through-ring shuttling occurs where one macrocycle can pass through the cavity of the other. This translation enables both macrocycles to move between both binding sites, increasing the flexibility of the rotaxane (Figure 4E→Figure 4A).

When more flexible threads are used, secondary interactions between macrocycles and the thread can impact switching kinetics in surprising ways. In a study by Jeppesen and Flood, [2]rotaxanes (Figure 14D) were composed of the CBPQT^4+^ macrocycle and flexible PEG-based thread. The rotaxane included the redox-active tetrathiafulvalene (TTF) and two oxyphenylene binding sites (Andersen et al., 2014). Methyl groups were introduced on the TTF station to serve as kinetic barriers. Upon oxidation to the TTF^2+^ dication, the CBPQT^4+^ is switched to the oxyphenylene station. Counterintuitively, the longer linkers were shown to have faster rates of switching relative to their shorter counterparts. Since the longer PEG chains interact better with the CBPQT^4+^ macrocycle, the glycols were believed to help the CBPQT^4+^ overcome the methyl-based steric barriers. This effect reduced the barrier to shuttling and caused an increase in reaction rate (Figure 4C→Figure 4A). When the macrocycle was switched back from the oxyphenylene station to the TTF station, the longer linkers behaved as anticipated and slowed down the kinetic rate (Figure 4A→Figure 4C). This study exemplifies how flexible chains can form unexpected interactions that greatly impact how the system operates. Working with more rigid components may lead to more controllable machines.

## Translating Molecular Designs Into Bulk Materials

Bulk materials are used to construct many of the technologies we interact with in our daily lives. New materials are always of interest for a wide array of applications including 3D printing inks (Li et al., 2019) and robotics (Wehner et al., 2016; Shih et al., 2020). One potential avenue to create these materials makes use of molecular designs that are translated into amorphous (soft) or crystalline (hard) materials. Here, we highlight a few promising areas where rigidity and flexibility are programmed into rotaxane designs and translated to material properties at the macroscale.

**FIGURE 15:**
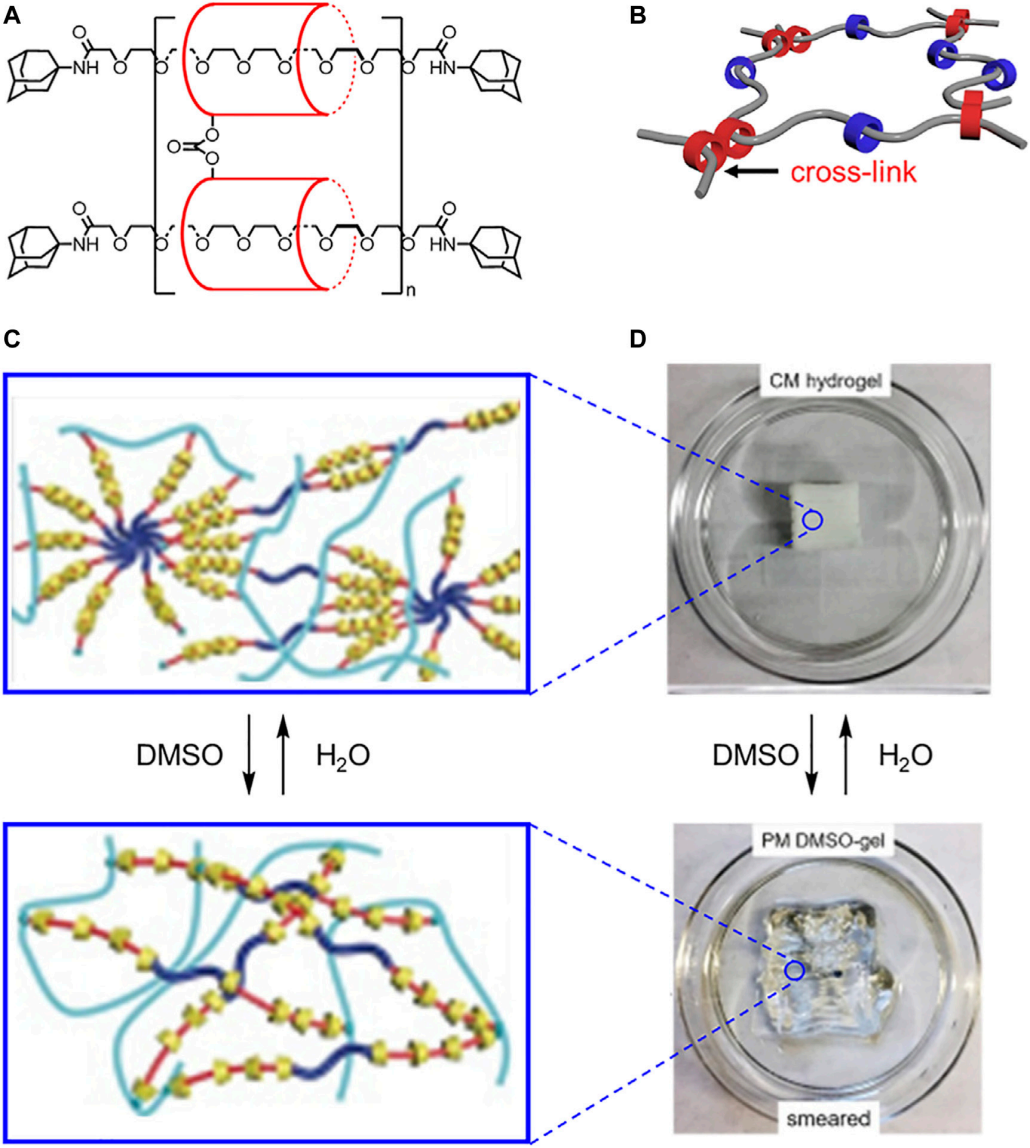
**(A)** Structure and **(B)** model of slide ring materials with cross-linked cyclodextrins (red) and free cyclodextrins (blue). **(C)** Molecular cartoons and **(D)** 3D printed materials under different solvent conditions. Adapted with permission from Ref. (Lin et al., 2017). Copyright 2017 John Wiley and Sons.

Slide ring materials use linked macrocycles to form cross-linked polymer chains. Translational mobility associated with the sliding of macrocycles along polymer threads can be used to govern the flexibility of bulk materials. In 2001, Ito created cross-linked poly[*n*]rotaxanes using rigid cyclodextrin macrocycles and flexible polyethylene glycol threads (Figure 15A). (Okumura and Ito, 2001) In these systems, α-cyclodextrin macrocycles are covalently linked together by a carbonate ester to create a figure of eight topology (Figure 15B). The polyethylene glycol chains slide through the macrocycles like a pulley (Liu et al., 2017), thereby creating a molecule with translational flexibility (Figure 4B). The consequences of this flexibility are expressed at the macroscopic level where the bulk material can expand and contract. To toughen the hydrogel and improve its self-healing properties, crosslinks between the polyethylene glycol threads can be used (Figure 4B→Figure 4A). (Liu et al., 2021)

The mechanical properties of slide-ring materials can be further tuned based on the length of the flexible polyethylene glycol chain and the density of the rigid cyclodextrin macrocycles (Liu et al., 2017; Kato et al., 2015). When the glycol chains are longer (*M*_w_ > 3.5 × 10^5^), sliding is enhanced, which creates an extensible, flexible material (Liu et al., 2017; Kato et al., 2015; Bitoh et al., 2011). However, when the glycol chains are shorter (*M*_w_ < 3.5 × 10^5^), chain mobility is reduced, and the material undergoes deformations when stretched (Figure 4B→4A). (Karino et al., 2005; Kato et al., 2015) Similarly, when the ring density is lower, the rings can freely slide along the chain, and the material shows better extension (Kato et al., 2015; Tanaka et al., 2018). When the ring density is increased (Figure 4B →Figure 4D), the material is much more fragile.

These differences in mechanical properties are related to cyclodextrin entropy and chain sliding (Kato et al., 2015). Both the higher and lower density polyrotaxanes had sufficient room for the cyclodextrins to slide along the thread and access different translational co-conformations. However, the sliding of the polymer chain through the macrocycle pulleys limits macrocycle entropy. As a result, when more cyclodextrins are present on the thread, chain sliding becomes less favored, and stretching of the polyethylene glycol thread, i.e., chain elongation, becomes favored. The stretching of this covalent polymer network results in a more brittle material.

Building on the idea of molecular pulleys, Coskun and Choi showed that cyclodextrin polyrotaxanes can serve as elastic binders and extend the cycling associated with silicon microparticle anodes in lithium ion batteries (Choi et al., 2017). Silicon is desirable for Li-ion batteries on account of its large specific capacity (Choi and Aurbach, 2016), but silicon also displays large volume changes upon charge-discharge cycles, which ultimately leads to the pulverization of the nanoparticles (Wang et al., 2013). Previous elastic binders were composed of crosslinked polymers, but these ultimately failed to sustain the silicon’s volume changes. The polyrotaxane pulley system was anticipated to introduce a stress-release mechanism where a series of pulleys lower the force required to coalesce disintegrated silicon microparticles (Figure 4E→Figure 4B). The polyrotaxanes are composed of single α-cyclodextrin macrocycles and stoppered polyethylene glycol threads. To create the pulley system, some of the cyclodextrins are cross-linked to an elastic polyacrylic acid (PAA) binder. Upon applying a strain, the polyethylene glycol threads can move freely inside the macrocycles to help relieve the stress. The composite polyrotaxane binder retained 91% capacity after 150 cycles, while use of PAA alone retained just 48% capacity after 50 cycles. Overall, the translational freedom of the polyethylene glycol chains enabled the silicon nanoparticles to experience less stress during charge-discharge cycles, allowing the Li-ion battery to last more cycles.

In 2017, Ke harnessed covalent thread-thread cross-linking and inter-macrocycle interactions to create solvent-switchable 3D-printable materials (Lin et al., 2017). Ke created a polypseudorotaxane hydrogel (Figure 15C) composed of the rigid α-cyclodextrin macrocycles and flexible PEG-based threads terminated by alkenes. When the hydrogel passes through the nozzle of an extruder during additive printing, dynamic hydrogen bonds between hydroxyl groups on neighboring cyclodextrin macrocycles break (shearing) and reform (self-healing). The most successful polypseudorotaxane hydrogel had a high degree of α-cyclodextrin threading and excess cyclodextrin in the gel. This high threading ratio decreased ring motion during the printing, and the excess cyclodextrin is believed to interdigitate between the rotaxanes to help self-heal crystalline domains (Figure 4B→Figure 4D). This hydrogen-bonding network generates the object’s ability to hold a shape. Once the 3D object is printed, the glycol-based threads can be photochemically crosslinked together using terminal alkenes to form polyrotaxanes thereby fixing the final structure. The crosslinking enables the hydrogen-bonding network to be reversibly disturbed and re-established using solvent exchange (Figure 15D). Ke showed that DMSO causes the 3D printed object to smear (Figure 4D→Figure 4B), and water recovers the original shape (Figure 4B→Figure 4D). Building on this work, cyclodextrin density and mobility have been shown to impact the resulting material properties (Lin et al., 2021), which is consistent with findings from Ito (Kato et al., 2015).

## Perspectives and Outlook

A variety of factors contribute to rotaxane rigidity and flexibility. The relative pre-organization and shape-persistence of molecular building blocks (macrocycle and threads) give a starting point to think about rigidity imposed by the covalently bonded structure. Generally, we find that hosts and guests are rigidified when present in an interlocked architecture by introduction of stabilizing intermolecular contacts and destabilizing steric interactions. Examples include hydrogen bonding interactions between the macrocycle and thread (Figure 7B, Figure 10C, Figures 12A,B, Figure 14C, Figure 15), (Clegg et al., 1999; Lee et al., 2013; Young et al., 2014; Lin et al., 2017) π-π stacking between macrocycles (Figures 9B,C, Figure 10C, Figure 12A, Figures 14B,D), (Liu et al., 2016; Belowich et al., 2012; Gong et al., 2010; Gong et al., 2011; Gong et al., 2012; Belowich et al., 2010; Nygaard et al., 2007; Andersen et al., 2014) metal coordination (Figures 5C,D, Figure 6A, Figures 8A,D,E), (Li et al., 2018; Sleiman et al., 1995; Chambron et al., 1993; Collin et al., 2010; Baggi and Loeb, 2016) steric interactions between macrocycles (Figures 12C,D), (Lewis et al., 2016) and electrostatic repulsions (Figures 13, 14E). (Qiu et al., 2020a) This rigidification is almost ubiquitous. While creation of an interlocked molecule introduces potential for rotational and translational co-conformations that add degrees of flexibility to an otherwise rigid rotaxane (Figure 6B, Figure 14B), (Nygaard et al., 2007; Collin et al., 2010) these likely fail to compensate for the rigidity gained upon threading. Overall, both the covalent bonding, non-covalent interactions and steric contacts impact the conformational space and thus the overall rigidity of the macrocycle and thread in rotaxane architectures.

**FIGURE 16:**
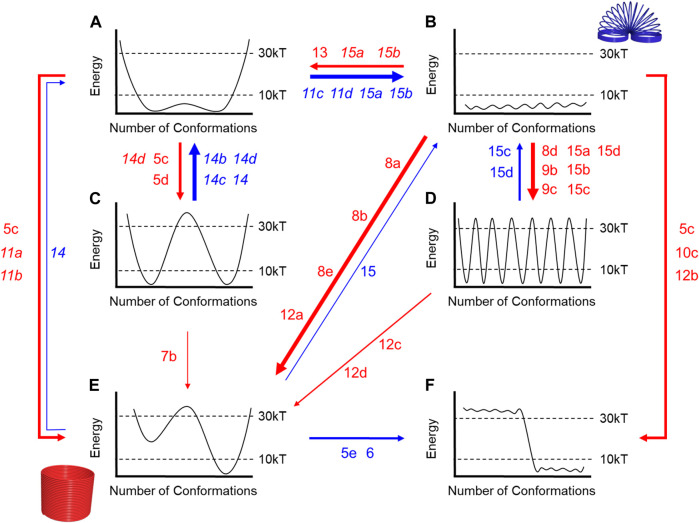
Flowchart annotated with Figure numbers that correspond to examples where the rotaxane either gets more rigid (red arrows) or more flexible (blue arrows) relative to its components. Thicker arrows represent more examples. Italics are used to indicate examples where two different rotaxane structures are being compared. Letters a-f correspond to the flow chart in Figure 4.

To help connect the changes in rigidity and flexibility outlined in the conformational landscapes to the threaded architectures examined in this *Review*, we created a map (Figure 16). Here, the numbers correspond to the figure call-outs for each molecular example. The red arrows indicate increasing rigidity while the blue is for enhanced flexibility. The thickness of the line scales with the number of examples. The majority of examples show rigidification with only a few showing elements of increased flexibility.

Looking forward, we see potential for rigidity and flexibility to play out in a variety of rotaxanes with both properties having a role to play. Polyrotaxanes and oligorotaxanes have multiple macrocycles on the thread, which introduce inter-macrocycle interactions that can be used to help engineer rotaxane rigidity and flexibility. These properties can also be transferred to the mechanical characteristics of materials. Examples include 3D printing inks, elastic binders, and hydrogels where high ring density or pervasive inter-macrocycle interactions led to harder materials while low ring density and fewer inter-macrocycle interactions led to softer materials. In these cases, both rigidity and flexibility could be beneficial depending on the design requirements of the application. At the same time, molecular switches and machines are a growing platform to perform work at the nanoscale (McTernan et al., 2020; Amano et al., 2021). Switches and machines with more rigid building blocks were shown to have fewer conformations and lower barriers to shuttling than their flexible counterparts, which suggests that more rigid designs could lead to more efficient and controllable machines. Thus, understanding the interplay between barriers and wells is critical to their successful operation. The study of these systems could also provide a platform to expand our understanding of the effects of barrier height on rigidity and flexibility.

## Conclusion

Rigidity and flexibility provide a new perspective for examining rotaxanes and their designs. Molecular rigidity and flexibility are not absolutes, rather they exist on a spectrum where fewer conformations and higher energy barriers are associated with rigidity, and their converse are associated with flexibility. We applied this framework to an analysis of rotaxanes and their pseudorotaxane and polyrotaxane relatives as well as their component macrocycles and threads. In the molecular building blocks, these ideas play out in preorganization, shape-persistence, collapsibility, and dynamics. Rotaxanes are more than just the sum of their parts. The threaded structures are universally rigidified even when starting with flexible components. However, multiple rotational and translational co-conformations can introduce flexibility along different degrees of freedom that are novel to the threaded topology. In some cases, however, flexible components produced unexpected outcomes suggesting that rigid components might be more reliable in molecular design. With the growing platform of rotaxanes in molecular switches, machines, and materials, the rigidity and flexibility of rotaxanes and their components will have important roles to play.
