# Corrigendum: Rigidity and Flexibility in Rotaxanes and Their Relatives; On Being Stubborn and Easy-Going

**DOI:** 10.3389/fchem.2022.953052

**Published:** 2022-08-04

**Authors:** Rachel E. Fadler, Amar H. Flood

**Affiliations:** Department of Chemistry, Indiana University, Bloomington, IN, United States

**Keywords:** conformations, flexible, host-guest chemistry, macrocycle, polyrotaxane, pseudorotaxane, rigid, rotaxane

In the original article, there were mistakes in some of the figures as published. The corrected figures are below.

In Figure 1F, the cyclodextrin was missing from the repeating unit.

**FIGURE 1:**
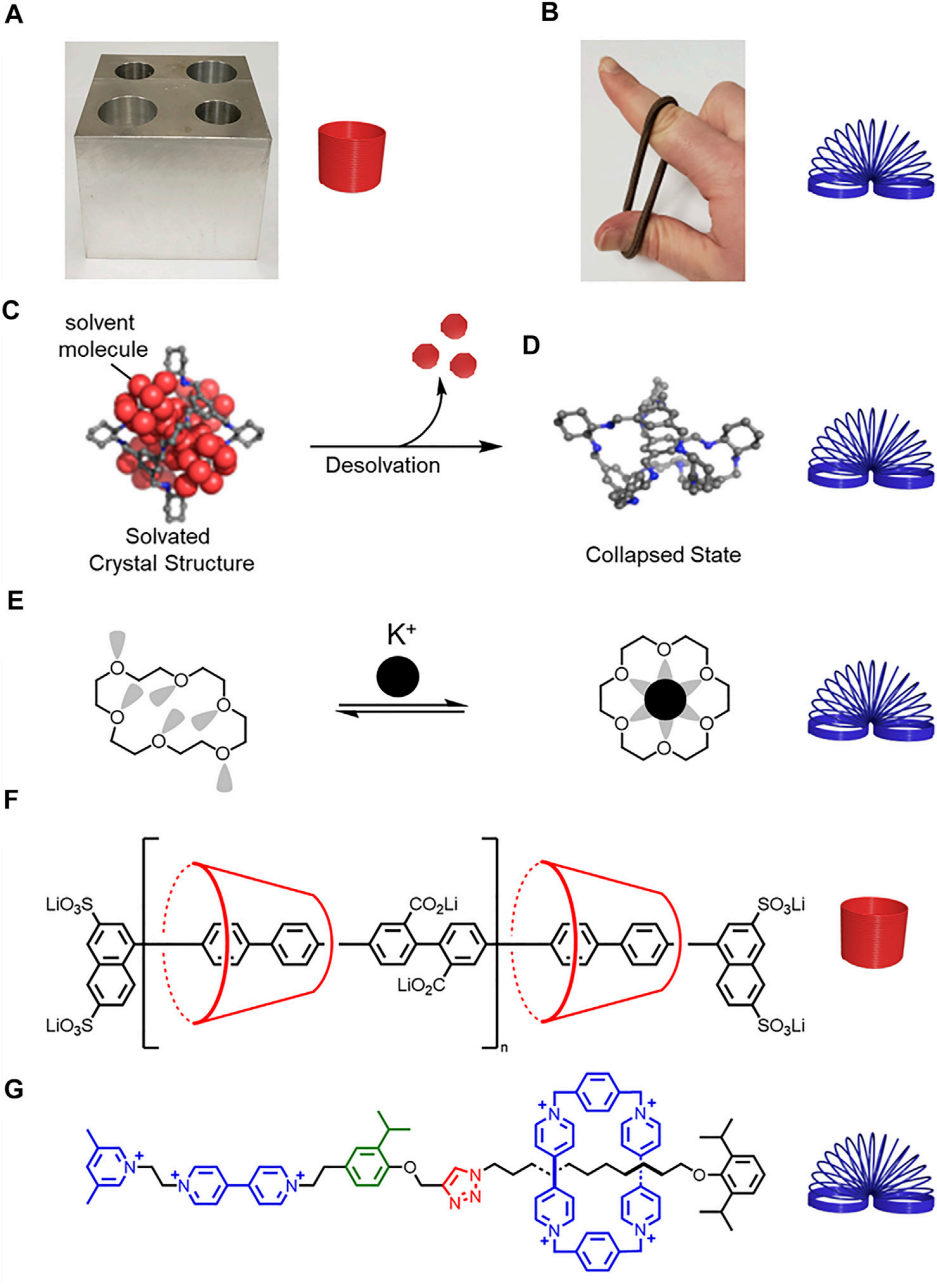
**(A)** Hard metal block and rigid spring. **(B)** Soft rubber band and flexible spring. **(C)** Schematic representation of a solvated molecular cage **(D)** collapsing upon desolvation. Adapted with permission from Ref. (**Liu et al., 2014**). Copyright 2014 American Chemical Society. **(E)** Structure of a collapsed crown ether that changes shape and rigidifies upon potassium complexation. **(F)** A more rigid polyrotaxane composed of cyclodextrin and a conjugated thread, and **(G)** a less rigid rotaxane composed of cyclobis (paraquat-p-phenylene) (CBPQT^4+^) and a thread composed of flexible alkyl chains and rigid aryl building blocks.

In Figures 5D,E, the organic substituents on the lower porphyrin ring were incorrect.

**FIGURE 5:**
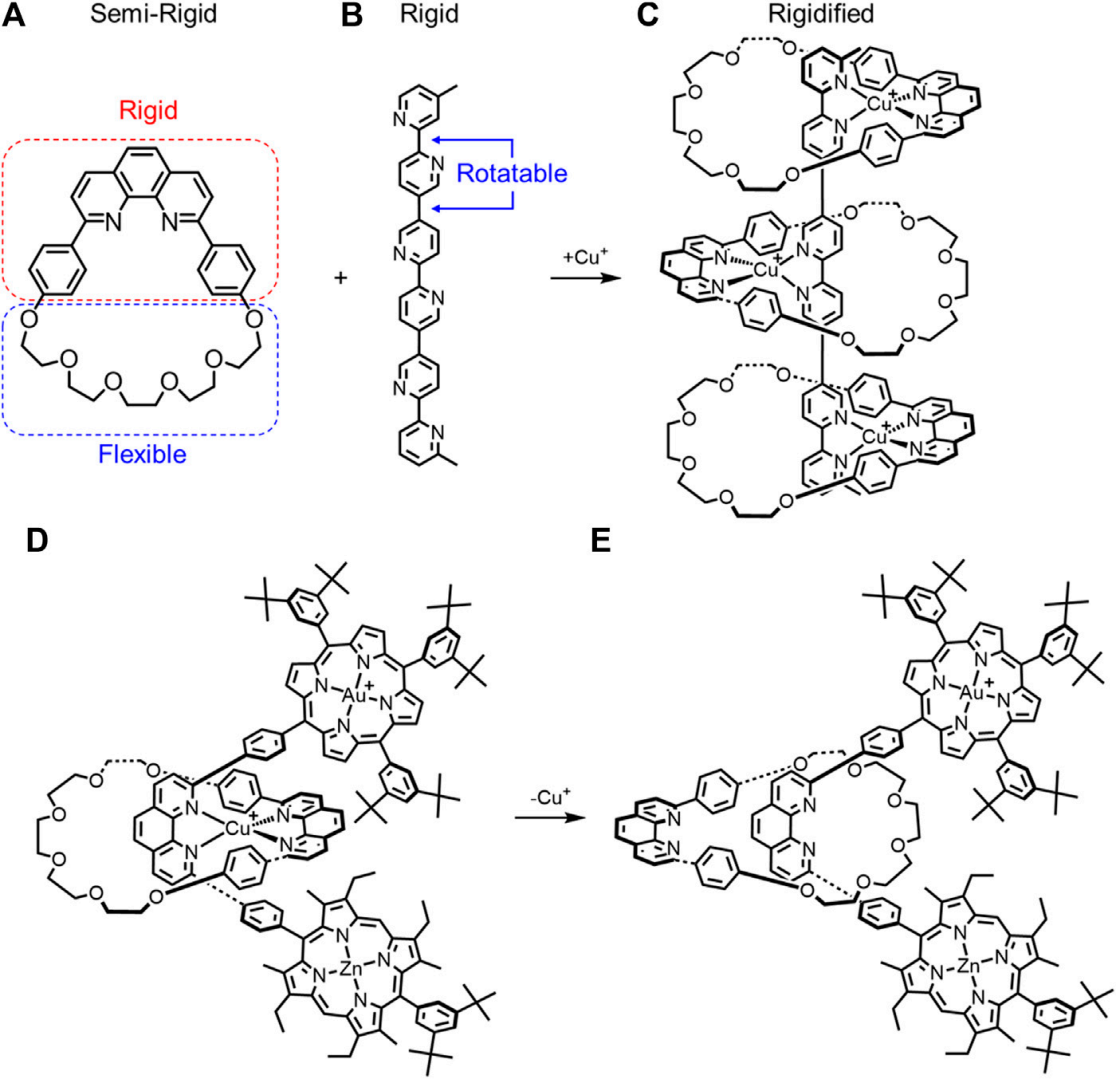
**(A)** Semi-rigid macrocycle and **(B)** rigid thread combine to make a **(C)** rigidified pseudorotaxane upon Cu^+^ complexation. A rotaxane composed of a semi rigid macrocycle and rigid thread have different macrocycle rotational conformations upon **(D)** metal complexation and **(E)** decomplexation.

In Figure 6, the chelating units on the thread consisted of a 4,7-phenanthroline and two pyridines rather than two 2,2′-bipyridines. In addition, the zinc coordinates to triazole in Figure 6B on both the top and bottom thread, not a pyridine nitrogen. Ar labels were also provided.

**FIGURE 6:**
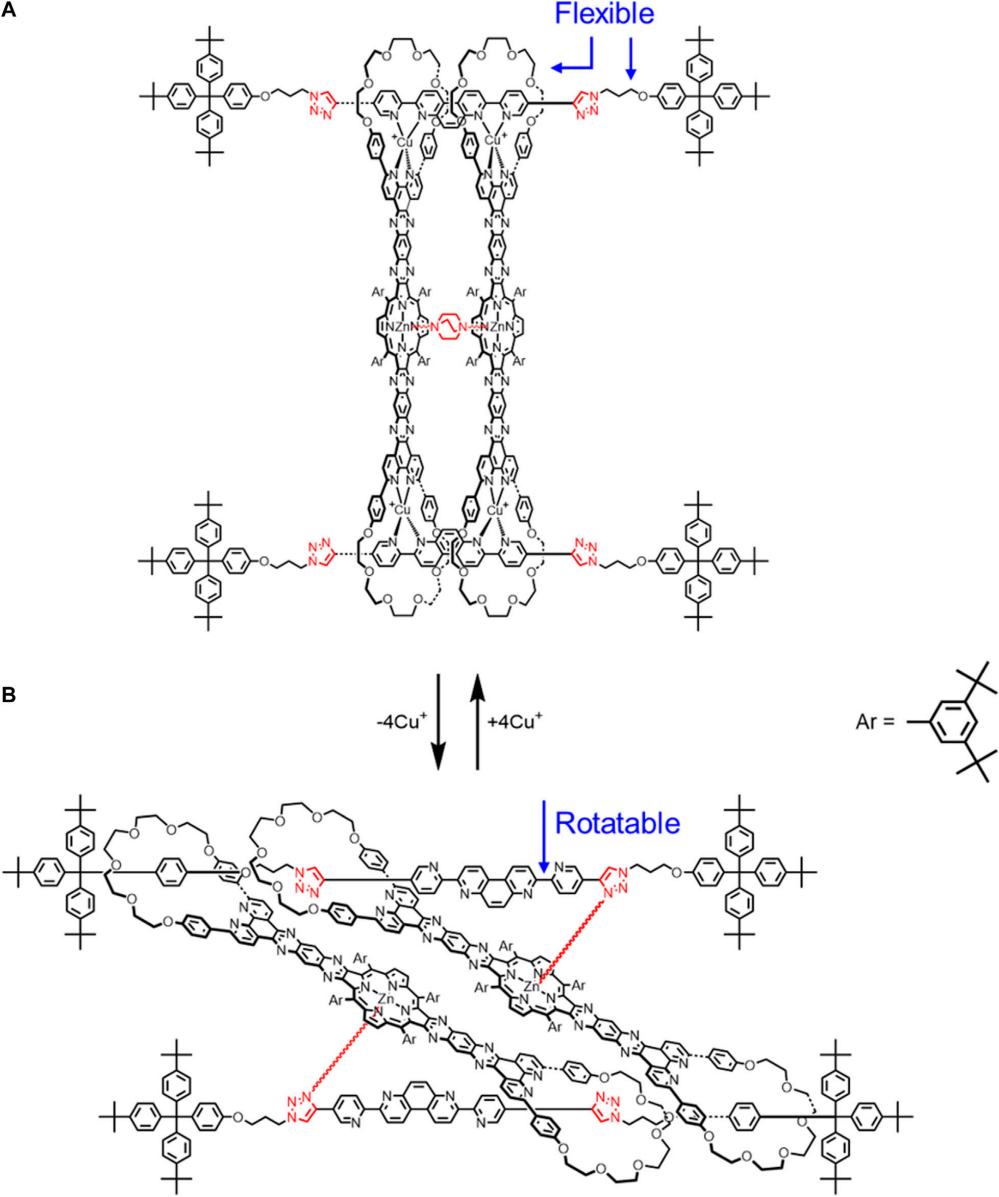
Cyclic [4]rotaxane switch with two translational states upon **(A)** Cu^+^ complexation and **(B)** decomplexation.

In Figure 13A, the methyl groups on the exterior pyridines were in the wrong position. The number of methylene carbons was fixed.

**FIGURE 13:**
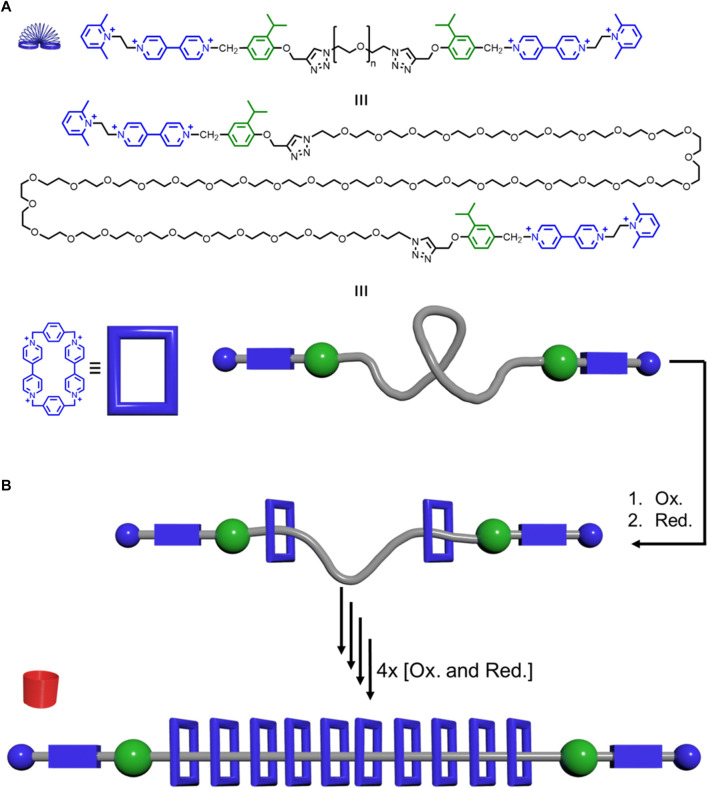
Precise polyrotaxane pump synthesizer: **(A)** Thread structure (top), elongated version (middle), and model together with the structure of the CBPQT^4+^ macrocycle (blue) and its model. **(B)** Pumping of the macrocycles onto the thread generates rotaxanes that get more rigid as more CBPQT^4+^ macrocycles are added.

In Figure 14D, the +2e^–^ and –2e^–^ were going in the wrong direction. The oxidized tetrathiafulvalene structure had a bridging double bond instead of a single bond.

**FIGURE 14:**
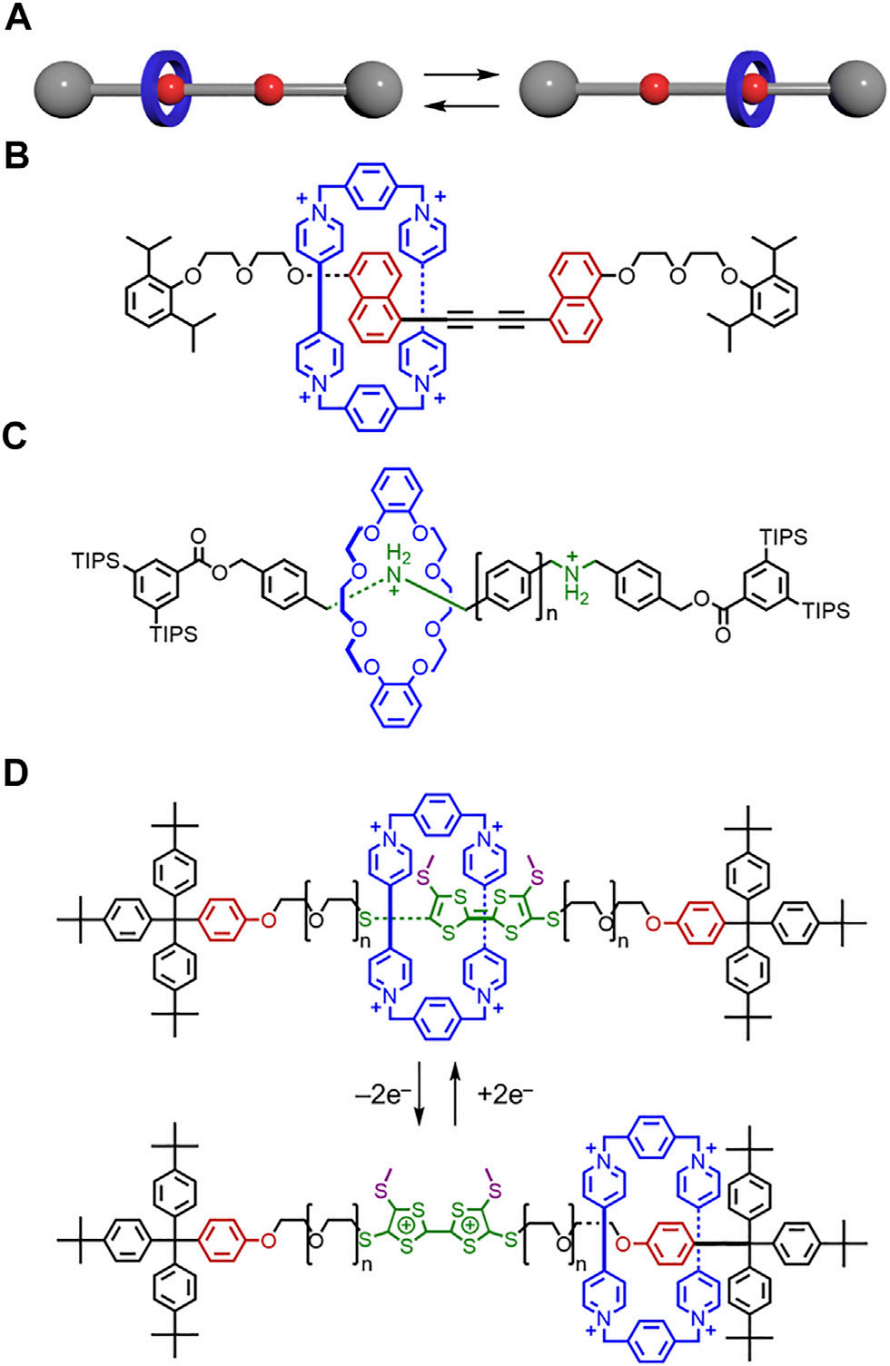
**(A)** Translational motion between isomers in a molecular shuttle. **(B)** Shuttle composed of a CBPQT^4+^ macrocycle and a rigid thread. **(C)** Shuttle composed of a crown ether macrocycle and a thread with rigid phenyl linkers. **(D)** Molecular switch composed of a CBPQT^4+^ macrocycle and a thread with flexible PEG linkers.

The H_2_O and DMSO solvent labels were flipped in Figures 15C,D.

**FIGURE 15:**
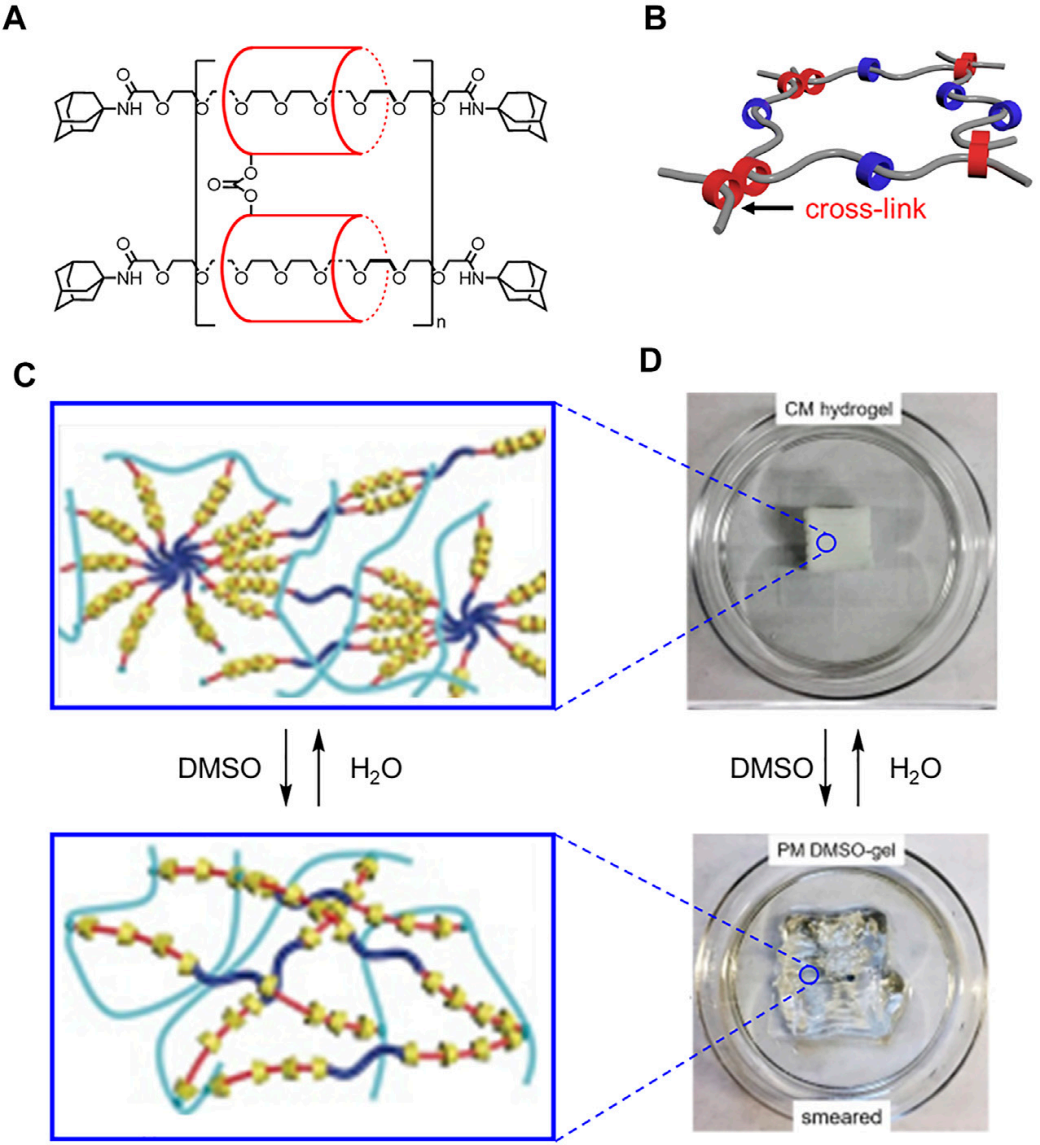
**(A)** Structure and **(B)** model of slide ring materials with cross-linked cyclodextrins (red) and free cyclodextrins (blue). **(C)** Molecular cartoons and **(D)** 3D printed materials under different solvent conditions. Adapted with permission from Ref. (**Lin et al., 2017**). Copyright 2017 John Wiley and Sons.

In the original article there was an error in the section **Translating Molecular Designs Into Bulk Materials**, page 18, last paragraph. The correct description of **Figure 4** is as follows:

“Ke showed that DMSO causes the 3D printed object to smear (**Figure 4D**→**Figure 4B**), and water recovers the original shape (**Figure 4B**→**Figure 4D**).”

The authors apologize for these errors and state that this does not change the scientific conclusions of the article in any way. The original article has been updated.

